# Integrating geospatial and environmental factors in colorectal cancer epidemiology: a regional study

**DOI:** 10.3389/fpubh.2025.1699870

**Published:** 2026-01-15

**Authors:** Dongqiang He, Xueliang Li, Fan Liu, Yalan Zhang, Shengrui Yang, Yang Zhao, Huijuan Cheng, Fan Zhang, Jie Liu, Yumin Li

**Affiliations:** 1 The Second Hospital of Lanzhou University, Lanzhou, China; 2 The Second Clinical Medical College of Lanzhou University, Lanzhou, China; 3 Gansu Province Key Laboratory of Environmental Oncology, Lanzhou, China; 4 Department of General Surgery, Key Laboratory of Digestive System Tumors of Gansu Province, The Second Hospital of Lanzhou University, Lanzhou, China

**Keywords:** colorectal cancer, environmental factors, geospatial, investigation of spatiotemporal variations, multivariate spatial analysis

## Abstract

**Background:**

Colorectal cancer poses a major health challenge in Gansu Province, being one of the top causes of both incidence and mortality among gastrointestinal cancers. This study aims to analyze the epidemiological patterns of colorectal cancer in Gansu Province, while exploring potential associations with environmental determinants.

**Method:**

We analyzed clinical records of all colorectal cancer cases from 2013 to 2023, retrieved from hospital information systems across 87 counties in Gansu Province, encompassing municipal, district, county, and township-level medical institutions. A thorough analysis was conducted employing various methods, including Joinpoint regression, spatial autocorrelation, spatiotemporal scanning, and Geo-detector analysis, using specialized software (Joinpoint 5.0, ArcGIS 10.8, and SaTScan). Our study explored the relationship between colorectal cancer incidence in Gansu Province and 14 Environmental factors.

**Result:**

The results indicate a steady rise in colorectal cancer incidence over the 11-year period and the highest age-standardized incidence rates of colorectal cancer occurred in Jinchuan, Chengguan, Suzhou, Baiyin, and Liangzhou districts, contrasting sharply with the significantly lower rates documented in Liangdang, Kang, Heshui, Huining, Zhengning, and Cheng counties. Spatial and spatiotemporal analyses identified several significant high- and low-risk clusters of colorectal cancer throughout Gansu Province, demonstrating both spatial and temporal variability in disease distribution. The Geo-detector indicated that colorectal cancer incidence was significantly linked to the distribution of climatic conditions (precipitation and temperature), ecological factor, and certain air pollutants. Multivariate spatial analysis was used to further explore the relationship between environmental factors and the incidence of colorectal cancer.

**Conclusion:**

Our research highlighted notable spatial variability in colorectal cancer incidence across Gansu Province, with geospatial and spatiotemporal analyses uncovering high-risk clusters and important environmental factors.

## 1 Introduction

Reports from the World Health Organization (WHO) indicate that cancer is now one of the top causes of death in nearly half of the world’s regions (1). Colorectal cancer (CRC) has become one of the most common cancers affecting the digestive system globally. Epidemiological research shows that its incidence and mortality rates are among the highest for cancers worldwide, with a steady increase over time. The GLOBOCAN database from the International Agency for Research on Cancer (IARC) reveals a significant 38% rise in new cases from 2007 to 2017, with annual diagnoses increasing from 1.3 million to 1.8 million during that period (2). In 2022, CRC was responsible for 1.93 million new cases and 904,000 deaths globally, making up 9.64% of all new cancer diagnoses and 9.28% of total cancer-related deaths (3). China faces a particularly heavy burden from CRC, with some of the highest incidence and mortality rates in the world, ranking as the second most common cancer in the country. Recent decades have seen a troubling rise in both the occurrence of the disease and death rates. In 2022, China reported 517,100 new cases (26.84% of the global total) and 240,000 deaths (26.67% worldwide), highlighting the urgent need for improved prevention and control strategies to tackle this escalating public health issue (4, 5).

Extensive epidemiological studies have increasingly shown that environmental exposures influenced by behavior are key factors in human cancer development (6). Certain environmental elements with epigenetic effects, such as behavioral habits, diet, and pollutants from chemicals and industries, can alter the structure of human chromatin, affecting gene expression and potentially leading to cancer (7). Research has indicated that factors like smoking, high cholesterol, obesity, alcohol use, metabolic syndrome, ulcerative colitis, sedentary lifestyle, low vitamin D levels, high consumption of red meat, and sugary beverages may be linked to CRC (8–10). It is also suggested that air pollution could contribute to inflammation and CRC by affecting the intestinal redox lipome and microbiome (11, 12). Geoinformatics and spatial epidemiology play a crucial role in presenting and communicating health data, such as cancer incidence and mortality, to enhance cancer prevention and control efforts (13). Given the diverse climate and geography of Gansu Province, using geographic and spatiotemporal analytical methods to explore patterns of CRC incidence is both necessary and methodologically sound. Geodetectors are a commonly used tool for examining spatial heterogeneity and are vital for investigating the link between environmental factors and CRC (14).

This study combines traditional statistical methods with spatial and spatiotemporal analytical techniques to create a multi-scale assessment framework for systematically evaluating the spatial distribution and temporal changes of standardized CRC incidence rates in Gansu Province, analyzed at both provincial and county levels. By employing geographic detector modeling, we identified the mechanisms linking various environmental indicators to CRC risk. The results provide a solid evidence base for developing targeted prevention and control strategies for CRC in specific regions.

## 2 Methods

### 2.1 Data sources and analytical cohort

This study was based on colorectal cancer registry data from Gansu Province, China, covering the period from 2013 to 2023. The data were obtained from a multi-source surveillance system comprising 262 hospitals across all 87 counties and districts of the province, with an initial identification of 85,652 records. And all data in this study were collected and processed in strict accordance with the *Guidelines for Cancer Registration in China*. Data quality and completeness were ensured through active follow-up, logical error checking, and cross-validation across multiple data sources, guaranteeing overall reliability. Data cleaning and case selection followed a rigorous multi-step procedure, as detailed in Supplementary Figure 1. Firstly, given the multi-institutional nature of the data sources and the extended time span, duplicate reports were common. Therefore, the primary and most critical step was automated deduplication based on unique national identification numbers. This process removed the majority of duplicate entries (*n* = 58,135), resulting in 27,517 uniquely confirmed cases.

Subsequently, these 27,517 unique cases underwent manual review to verify the completeness of their demographic information. The inclusion criteria required that each record contain sex, year of diagnosis, and a registered residential address specified at the county or district level to ensure feasibility for subsequent spatial analyses. Upon verification, all deduplicated cases met these completeness requirements, and thus no additional cases were excluded at this stage. The final analytical cohort consisted of 27,517 unique cases with complete information, representing 32.1% of the initial records. The demographic composition was 57.1% male and 42.9% female. And the rigorous data processing procedures ensured high data quality and broad representativeness, providing a robust foundation for the subsequent spatial epidemiological analyses.

### 2.2 Environmental data collection and processing

This study incorporated various environmental data from Gansu Province’s 87 counties (2003–2013), which included ecological factors Normalized Difference Vegetation Index (NDVI), precipitation, humidity, mean temperature, air pollutants [PM₁₀, sulfur dioxide (SO₂), nitrogen oxides (NOₓ), ozone (O₃), carbon monoxide (CO), organic carbon (OC)], and greenhouse gases [methane (CH₄), ammonia (NH₃), CO2 emissions, greenhouse gas composite (GHG)], socioeconomic factors (GDP per capita, industrial outputs, hospital beds/10,000 population). The above data were obtained from the Resource and Environment Science and Data Center, National Geographic Information Public Service Platform, National Qinghai-Tibet Plateau Scientific Data Center Platform, Gansu Statistical Yearbook, China County Statistical Yearbook, and other sources. And all datasets were spatially standardized to 1-km^2^ grids and temporally aligned to annual resolutions using Geographic Information System (GIS) techniques to ensure consistency for geospatial analysis. Regarding the aggregation of environmental data from 1-km grids to county-level administrative units, we applied the “centroid assignment” principle for spatial matching, meaning that only grid cells whose centroids fall within a county boundary were included in the calculation. For continuous variables (such as NDVI, pollutant concentrations), we calculated the arithmetic mean of all valid grids within each county. For cumulative variables (such as GDP), we summed the grid values, while for ratio variables (such as hospital bed density), population-weighted averages were used. The description of the statistical charts for environmental factors is as described in Supplementary Table 1. All processing was conducted using ArcGIS 10.8, and counties with more than 10% missing grid data were specially flagged. These procedures ensure both the accuracy and reproducibility of the spatial aggregation process. Subsequently, we calculated the average exposure levels of these environmental factors during the period 2003–2013, and then explored their associations with colorectal cancer incidence observed over the 2013–2023 decade.

### 2.3 Statistical methods

Based on detailed residential address information, each patient’s place of residence was accurately assigned to a specific county, with an inclusion criterion requiring a minimum of 10 years of residency in that county. The crude incidence rates and age-standardized incidence rates (ASIRs) of colorectal cancer for all counties in Gansu Province were calculated using R4.5.0 software. To ensure the accuracy of the population denominators, crude incidence rates were derived using data from the 2022 National Census and the Gansu Statistical Yearbook, with the average population between 2013 and 2023 used as the denominator (unit: persons). The ASIRs were standardized according to the age distribution of the 2000 national census population of China. Age groups were categorized based on the demographic data, with individuals aged 85 and older grouped in five-year intervals. The age-standardized incidence rate (ASIR) was calculated for each age group separately. Additionally, we conducted linkage regression analysis to assess the temporal trends of CRC incidence rates in Gansu Province, as well as for both men and women. Trend surface correlation analysis was performed with incidence data and regional geographic correlation data, and using ArcGIS Pro 3.4.2, we illustrated the overall incidence rates by region and their annual distribution in Gansu Province from 2013 to 2023. We also calculated the median and mean ages of patients for each year and demonstrated the trends of these indicators over time, including changes in median and mean ages for both males and females. Patients were then categorized into three groups: under 35, 35–59, and 60 and older, and the trends of their respective percentages each year were analyzed. Furthermore, we examined the trends of the percentage of each age group for both men and women.

### 2.4 Spatial epidemiological analysis

We employed spatial autocorrelation analysis to examine the spatial distribution patterns of colorectal cancer incidence in Gansu Province. County-level administrative boundary data were integrated with colorectal cancer incidence rates to construct multiple spatial weight matrices for sensitivity analysis, including Queen contiguity (shared borders or vertices), Rook contiguity (shared borders only), distance-based threshold matrices derived from centroid distances (using the median, 75th percentile, and 90th percentile thresholds), and K-nearest neighbor matrices (K = 4, 6, 8). For “island” counties lacking natural neighbors, the nearest-neighbor method was applied to ensure spatial continuity. Global spatial autocorrelation was evaluated using Global Moran’s I to assess overall clustering, with 99 Monte Carlo permutations performed to test statistical significance, and the Global Getis-Ord General G statistic (Getis-Ord General G) calculated as a complementary measure. Local Indicators of Spatial Association (LISA) was analyzed using Local Moran’s I to identify high–high, low–low, high–low, and low–high clusters, and the Getis-Ord Gi* statistic to detect significant hot and cold spots, with multiple comparison corrections applied. Sensitivity analyses across different spatial weight matrices were conducted to assess the robustness of the results and ensure the reliability of the spatial patterns observed.

To identify statistically significant clusters, we used SaTScan™ 10.3 software, applying its spatial scan statistic. The maximum spatial scanning window was set to 25% of the at-risk population, and the maximum temporal window was defined as 50% of the study period. We employed the discrete Poisson probability model to carry out both purely spatial and spatiotemporal scan analyses to identify areas with increased or decreased risk. In addition, following your suggestion and that of another reviewer, we clearly defined the age groups as young (<35 years), middle-aged (35–59 years), and older population (≥60 years), and subsequently conducted the spatial analysis based on these classifications. For each cluster identified, we calculated the relative risk (RR) and corresponding *p*-values to assess statistical significance.

### 2.5 Geographical detector analysis of environmental risk factors for CRC

(1) A total of 20 potential environmental factors linked to CRC were identified from extensive databases, such as the China Statistical Yearbook and the Gansu Provincial Yearbook. Continuous variables were categorized using the natural breaks method (Quartile Method) to reduce variance within categories while enhancing differences between them.
(2) The factor detector was utilized to measure the explanatory power of each environmental variable regarding the spatial variation in incidence rates, as indicated by the q-statistic: $q=1-\frac{{?}_{h=1}^{L}N_{h}s_{h}^{2}}{Ns^{2}}$. In this context, N_h and σ_h^2^ represent the sample size and within-stratum variance for the h-th stratum, respectively, while N and σ^2^ refer to the total sample size and overall variance for the entire area. The significance of this formula lies in its capacity to evaluate how well an environmental variable explains the spatial distribution of the target variable. A significant reduction in within-stratum heterogeneity through stratification will result in a much smaller within-stratum variance (σ_h^2^) compared to the total variance (σ^2^), pushing the q-value closer to 1. In contrast, if stratification does not effectively reduce heterogeneity, the q-value will be near 0, indicating weak explanatory power. A q-value below 0.1 suggests a minimal contribution, while a value above 0.4 indicates a strong influence of the independent variable on incidence rates. Furthermore, a *p*-value less than 0.05 confirms statistical significance.
(3) The interaction detector examines how two independent variables collectively affect a dependent variable by comparing their combined impact to their individual effects, identifying five types of interactions: non-linear weakening (combined effect is less than the sum), single-factor non-linear weakening (between individual effects), bilinear enhancement (combined effect equals the sum), independent (matches the stronger factor), and non-linear enhancement (combined effect exceeds the sum). This method is particularly useful in spatial epidemiology for revealing complex relationships between environmental factors that influence disease distribution. Statistically significant results (*p* < 0.05) provide strong evidence of interaction patterns that can uncover underlying disease mechanisms. By quantifying non-linear relationships beyond simple additive effects, this approach offers valuable insights into multifactorial disease causation and aids in developing targeted public health strategies. Futhermore, All reported interaction *q*-values were assessed using 99 permutation tests to obtain the corresponding *p*-values.

### 2.6 Multivariable spatial analysis

This study adopts a spatial econometric framework to examine and quantify the potential spatial effects among the research variables. Based on the results of the geographical detector interaction analysis, we first selected the most influential variables to construct an Ordinary Least Squares (OLS) model as a baseline. To address the estimation bias that may arise from neglecting spatial dependence in the OLS model, we further estimated a series of spatial models, including the Spatial Lag Model (SLM), Spatial Error Model (SEM), Spatial Durbin Model (SDM), and the General Nested Spatial (GNS) model. This multi-model approach allows for a comprehensive capture of spatial autocorrelation that may exist either in the dependent variable spillovers or in the error term.

Model selection followed strict statistical criteria. We systematically compared the Akaike Information Criterion (AIC) and the Bayesian Information Criterion (BIC) values across models and examined the statistical significance of the spatial autoregressive coefficients to evaluate model performance. Ultimately, following the principle of minimizing AIC and BIC values, and considering the significance of spatial parameters, we identified the optimal model specification. Based on this selected model, we conducted an in-depth analysis and interpretation of the causal relationships and underlying spatial mechanisms among the variables.

## 3 Result

### 3.1 Eidemiological trend of CRC incidence in Gansu Province

#### 3.1.1 Relationship between age and CRC incidence in Gansu Province

**Figure 1:**
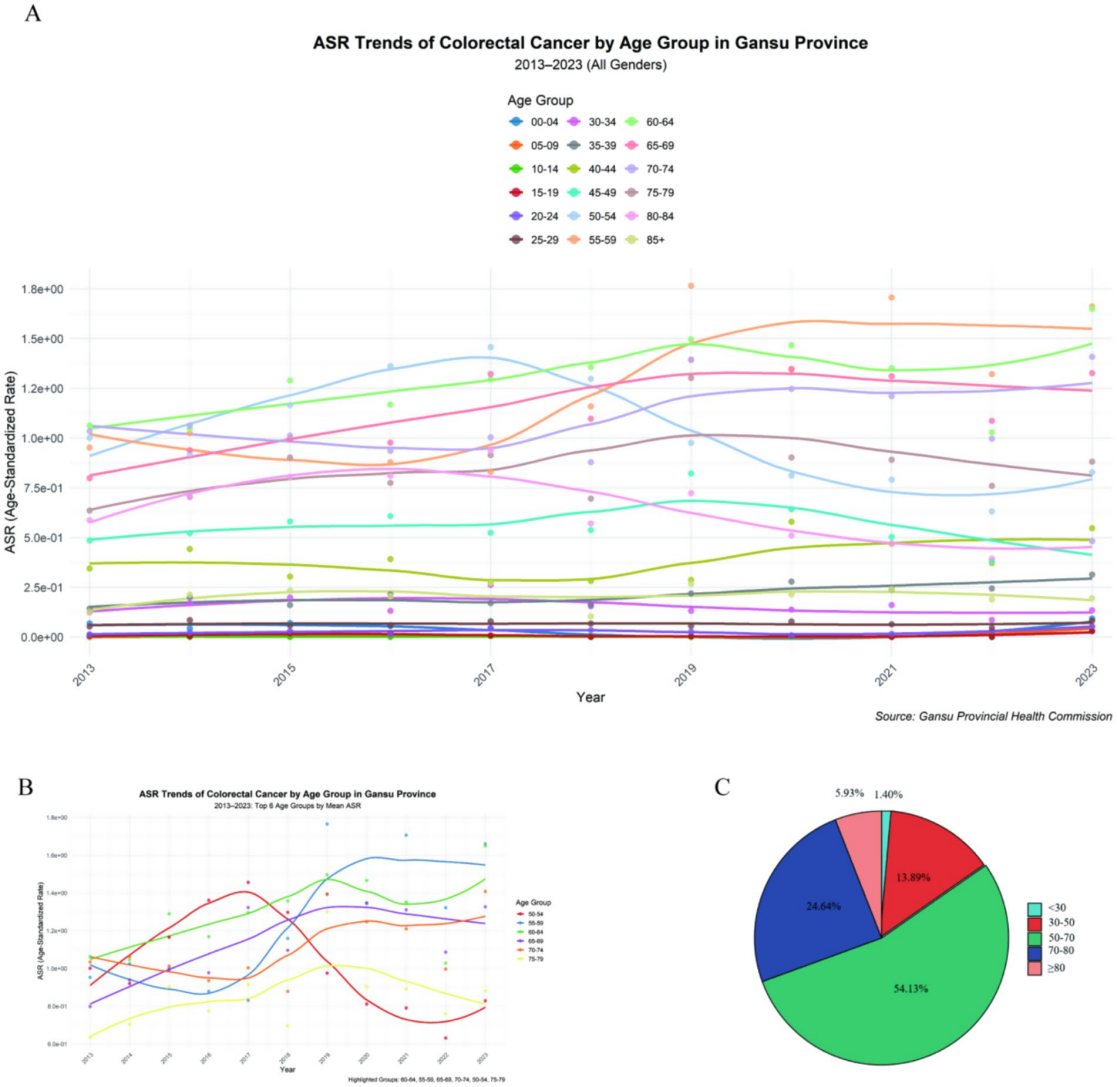
Age composition of included CRC patients. **(A)** Differences among various age groups. **(B)** A closer look at this important age group. **(C)** Age distribution map.

This research ultimately analyzed 27,517 CRC cases with complete age data. As shown in Figure 1A, patients were divided into 18 age categories, ranging from 0–4 years to over 85 years, highlighting significant differences among various age groups. The age-specific incidence analysis (Figure 1C) indicated a higher prevalence of the disease in older individuals (aged 50 and above) compared to younger ones. Specifically, the incidence rates were notably higher in the 50–80 age range (Figure 1A). A closer look at this important age group (Figure 1B) revealed six subcategories, with consistent increases in incidence rates for those aged 55–74, except for the 50–54 and 75–79 age groups.

**Figure 2:**
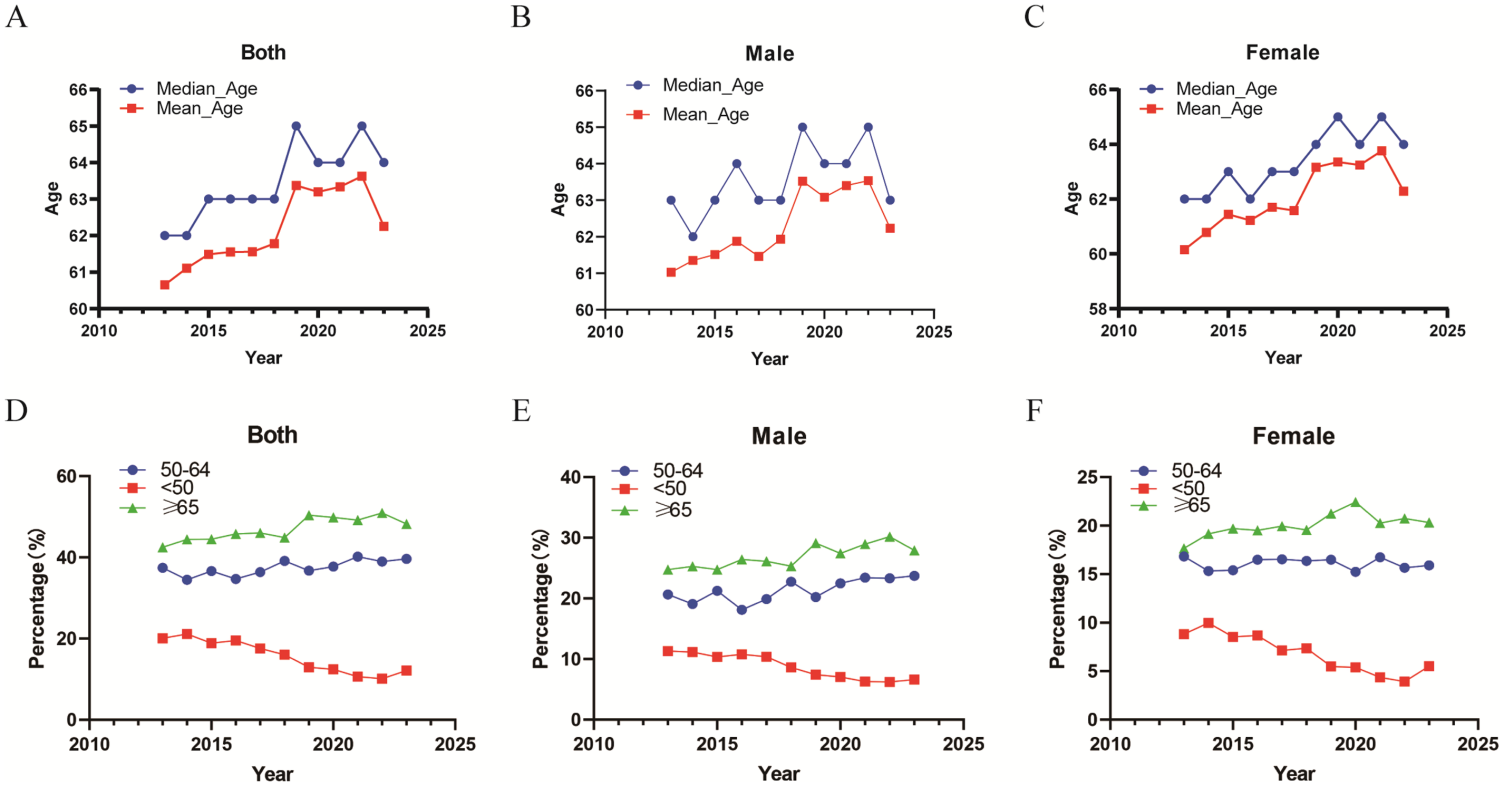
Trends in mean age, median age, and age proportions of included CRC patients. **(A)** Mean and median age of all patients over time. **(B)** Mean and median age of male patients over time. **(C)** Mean and median age of female patients over time. **(D)** Trends in age proportions of all patients over time. **(E)** Trends in age proportions of male patients over time. **(D)** Trends in age proportions of all patients over time. **(E)** Trends in age proportions of male patients over time. **(F)** Trends in the proportion of female patients’ ages over time.

Figures 2A,D outlines the epidemiological features in Gansu Province, where the average and median ages at diagnosis were 62.17 and 63 years, respectively. Longitudinal analysis showed a steady rise in both the mean and median ages from 2013 to 2023 (Figures 2B,C,E,F). Additionally, our findings indicated an increasing percentage of cases in patients over 50 years old, while there was a corresponding decrease in cases among those under 50.

#### 3.1.2 Gender differences in CRC incidence in Gansu Province

**Figure 3:**
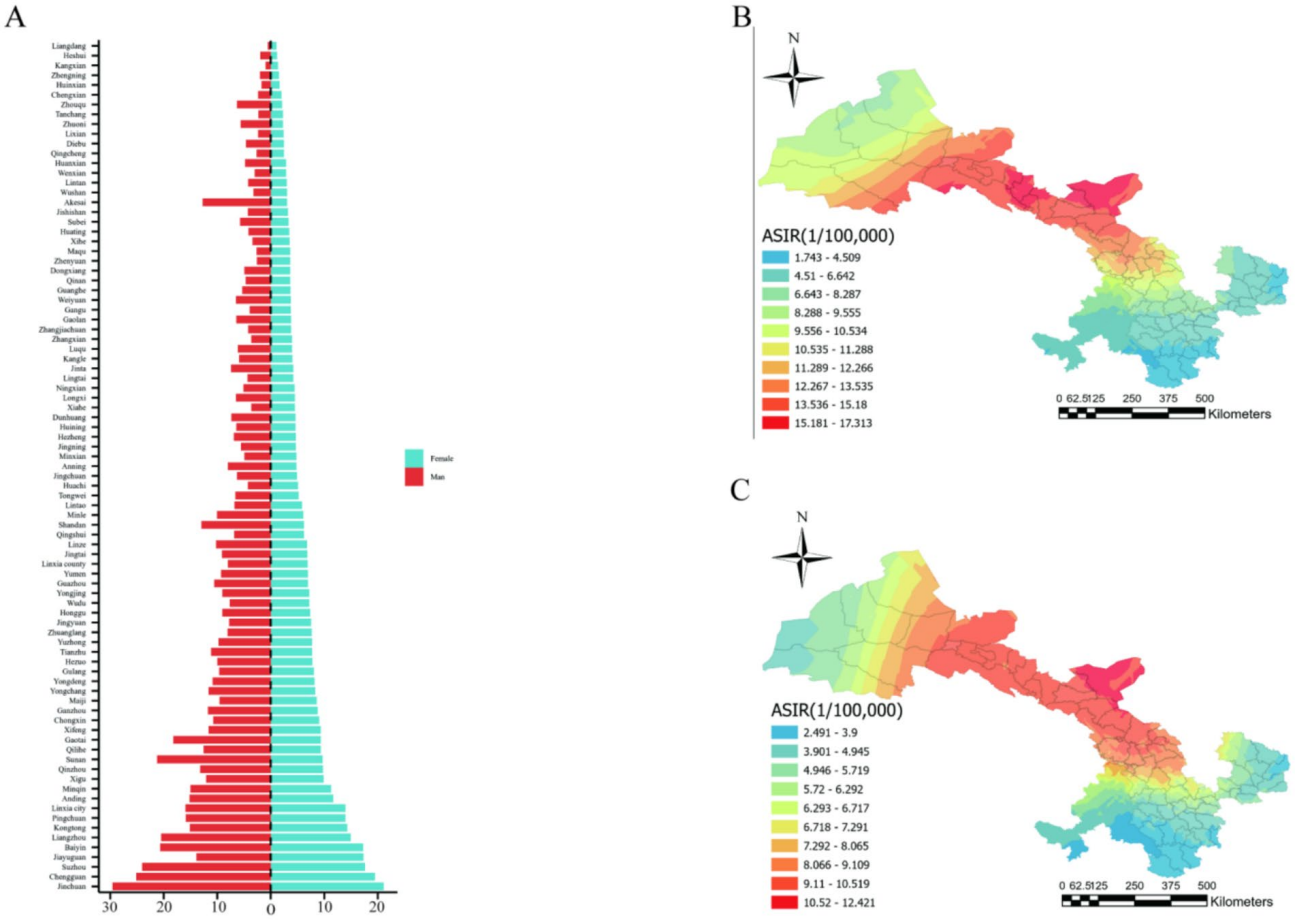
Gender differences in CRC incidence in Gansu Province. Gender differences in colorectal cancer incidence in Gansu province. **(A)** ASIR for colorectal cancer by gender in different counties (1/100,000). **(B)** Geographical distribution of male patients. **(C)** Geographical distribution of female patients.

Our research involved 15,724 male and 11,794 female patients with CRC, resulting in a male-to-female incidence ratio of about 1.33:1. We also computed the age-standardized incidence rates (ASIR) for both genders in each county of Gansu Province and illustrated the gender-specific comparisons in Figure 3A. According to the most recent national census data, Jinchuan had the highest incidence rate for males and females. Conversely, Liangdang exhibited the lowest incidence rates for both sexes (Figures 3B,C).

#### 3.1.3 Regional distribution of CRC in Gansu Province

**Figure 4:**
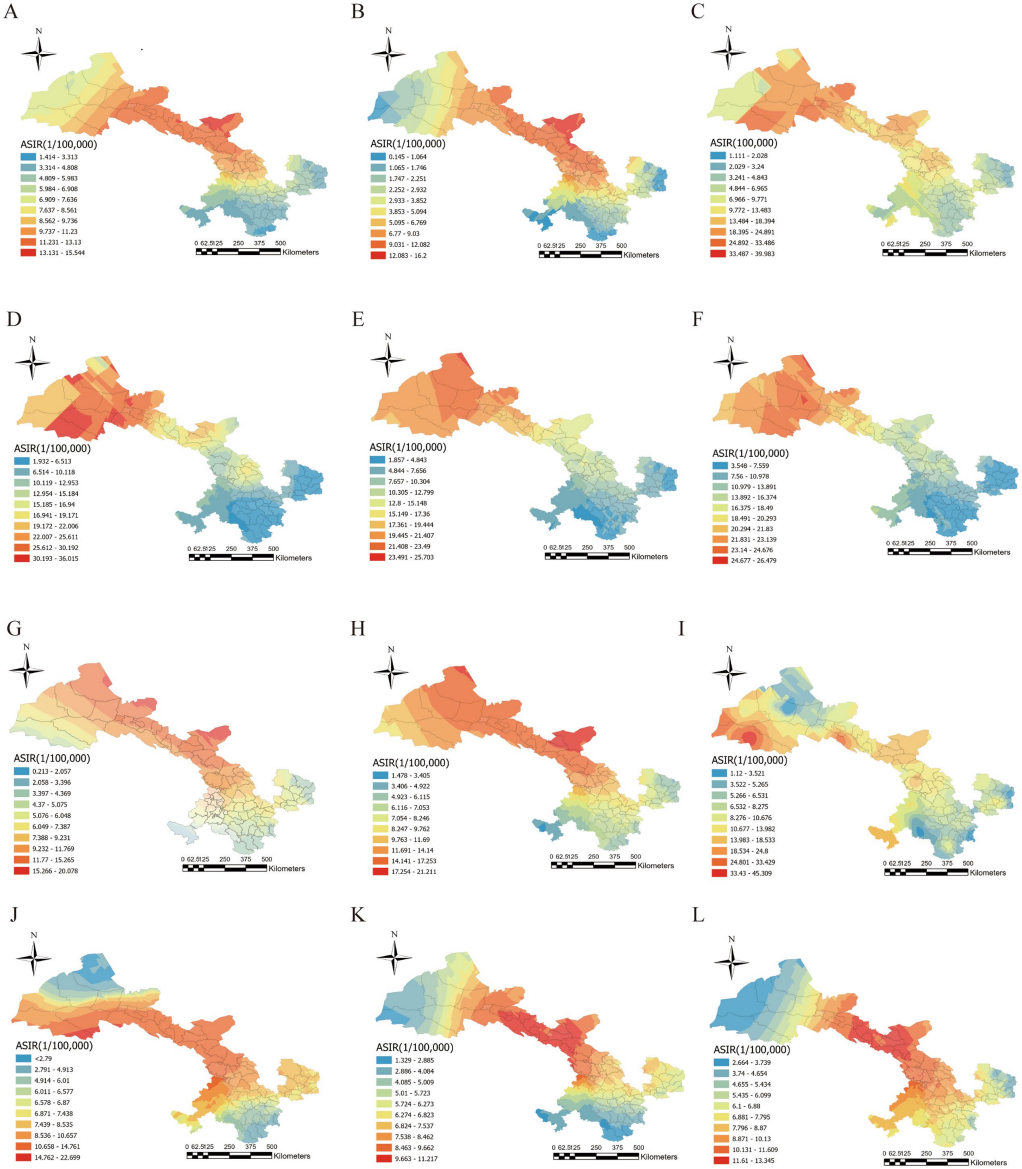
Regional distribution of CRC in Gansu Province. **(A)** Overall distribution of CRC ASIR in different counties and districts in Gansu Province. **(B–L)** Geographic distribution of age-standardized incidence rates (ASIR) for CRC in different counties and districts of Gansu Province in 2013–2023: **(B)** Geographic distribution of age-standardized incidence rates (ASIR) for colorectal cancer in different counties and districts of Gansu Province in 2013. **(C)** Geographic distribution of age-standardized incidence rates (ASIR) for colorectal cancer in different counties and districts of Gansu Province in 2014. **(D)** Geographic distribution of age-standardized incidence rates (ASIR) for colorectal cancer in different counties and districts of Gansu Province in 2015. **(E)** Geographic distribution of age-standardized incidence rates (ASIR) for colorectal cancer in different counties and districts of Gansu Province in 2016. **(F)** Geographic distribution of age-standardized incidence rates (ASIR) for colorectal cancer in different counties and districts of Gansu Province in 2017. **(G)** Geographic distribution of age-standardized incidence rates (ASIR) for colorectal cancer in different counties and districts of Gansu Province in 2018. **(H)** Geographic distribution of age-standardized incidence rates (ASIR) for colorectal cancer in different counties and districts of Gansu Province in 2019. **(I)** Geographic distribution of age-standardized incidence rates (ASIR) for colorectal cancer in different counties and districts of Gansu Province in 2020. **(J)** Geographic distribution of age-standardized incidence rates (ASIR) for colorectal cancer in different counties and districts of Gansu Province in 2021. **(K)** Geographic distribution of age-standardized incidence rates (ASIR) for colorectal cancer in different counties and districts of Gansu Province in 2022. **(L)** Geographic distribution of age-standardized incidence rates (ASIR) for colorectal cancer in different counties and districts of Gansu Province in 2023.

As illustrated in Figure 4, this research utilized data from the CRC registry in Gansu Province from 2013 to 2023 to examine the overall spatial distribution of age-standardized incidence rates (ASIR) over the 11-year span, as well as annual geographic trends. We also computed the cumulative ASIR for each county in the province and compiled the total number of cases by region during the study period (Table 1). The results reveal a gradual movement of high-incidence areas from central and northern Gansu toward the east and south over time. Between 2013 and 2017, the highest incidence rates were mainly found in the central and northwestern regions of the province, including Jiayuguan City, Jinchuan District, and Suzhou District. Although the overall rise in incidence slowed from 2018 to 2023, several counties—such as Jinchuan, Chengguan, Suzhou, Baiyin, Liangzhou, Jiayuguan, Sunnan Yugur Autonomous County, Linxia City, Pingchuan District, Gaotai County, and Anding District—continued to report relatively high rates.

**Table 1:** Regional distribution of CRC patients by county in Gansu Province in 2013–2023.

County	Cases (11-year total)	Population (person-year)	Incidence (1/100,000)	ASIR (1/100,0000)	AAPC (%)
Province total	29,622	281,156,908	10.54	8.95	1.57
Chengguan	3,898	15,002,382	25.98	22.2857	−9.87
Qilihe	930	6,825,454	13.63	10.9987	−2.61
Xigu	637	4,215,711	15.11	11.0162	−3.85
Anning	236	3,792,114	6.22	6.41026	19.33
Honggu	167	1,542,035	10.83	8.24393	13.82
Yongdeng	548	4,196,805	13.06	9.53297	14.22
Gaolan	120	1,479,849	8.11	5.12388	35.22
Yuzhong	524	5,044,763	10.39	8.79911	9.39
Jiayuguan	512	2,948,450	17.37	15.3893	−24.45
Jinchuan	817	2,652,662	30.8	25.5691	−13.15
Yongchang	299	2,384,267	12.54	9.98156	3.44
Baiyin	831	3,415,277	24.33	19.0292	−20.31
Pingchuan	342	2,150,126	15.91	14.8792	−8.93
Jingyuan	415	4,713,163	8.81	7.66098	16.41
Huining	345	5,440,533	6.34	5.54879	3.03
Jingtai	226	2,391,542	9.45	8.01298	10.25
Qinzhou	921	7,180,669	12.83	11.5962	−2.33
Maiji	660	6,133,099	10.76	9.19568	0
Qingshui	215	2,860,086	7.52	6.54806	17.53
Qinan	284	5,356,208	5.3	4.17979	46.65
Gangu	282	6,022,125	4.68	3.82009	32.35
Wushan	160	4,575,503	3.5	3.14475	40.29
Zhangjiachuan	124	3,021,421	4.1	4.06084	14.88
Liangzhou	2,183	10,650,678	20.5	17.8264	0.05
Minqin	457	2,414,650	18.93	13.2145	−5.78
Gulang	383	3,731,790	10.26	8.87325	27.47
Tianzhu	223	1,832,158	12.17	9.47377	6.38
Ganzhou	778	5,705,048	13.64	10.3274	16.14
Sunan	64	352,485	18.16	15.3211	12.58
Minle	231	2,340,725	9.87	8.09071	0.99
Linze	175	1,425,113	12.28	8.6099	22.2
Gaotai	266	1,529,117	17.4	13.902	−21.37
Shandan	211	1,758,825	12	9.64915	6.5
Kongtong	960	5,600,318	17.14	14.7514	−6.89
Jingchuan	220	2,879,813	7.64	5.64022	9.67
Lingtai	109	1,916,835	5.69	4.27986	12.97
Chongxin	115	1,056,532	10.88	9.93436	38.09
Zhuanglang	365	4,071,480	8.96	7.83505	1.29
Jingning	291	4,495,391	6.47	5.14553	−0.28
Huating	93	2,069,736	4.49	3.87809	−15.73
Suzhou	1,262	4,889,951	25.81	20.9775	0.69
Jinta	114	1,530,341	7.45	5.98085	−10.16
Guazhou	147	1,581,319	9.3	8.85772	−7.62
Subei	6	166,118	3.61	4.60904	−9.87
Akesai	6	118,357	5.07	8.39132	30.11
Yumen	177	1,691,482	10.46	8.2276	4.25
Dunhuang	152	2,059,567	7.38	6.08889	11.65
Xifeng	492	4,711,611	10.44	10.5275	22.68
Qingcheng	78	2,786,786	2.8	2.57921	23.46
Huanxian	150	3,362,240	4.46	3.92995	36.91
Huachi	67	1,342,322	4.99	4.64833	15.24
Heshui	31	1,584,014	1.96	1.57631	6.19
Zhengning	48	1,976,179	2.43	1.80125	18.1
Ningxian	284	4,236,312	6.7	4.80172	51.27
Zhenyuan	189	4,377,595	4.32	3.1008	27.71
Anding	842	4,713,776	17.86	13.4904	0.1
Tongwei	322	3,850,945	8.36	5.92917	18.48
Longxi	356	4,968,872	7.16	5.53523	28.53
Weiyuan	239	3,426,345	6.98	5.21753	7.15
Lintao	458	5,540,173	8.27	6.34129	9.96
Zhangxian	91	2,070,195	4.4	3.7868	16.37
Minxian	247	4,930,249	5.01	4.86057	7.07
Wudu	602	6,098,221	9.87	7.45291	48.92
Chengxian	68	2,687,492	2.53	2.19717	11.8
Wenxian	83	2,320,704	3.58	2.94571	9.14
Tanchang	86	2,970,767	2.89	2.27542	10.57
Kangxian	29	1,912,128	1.52	1.12115	−6.87
Xihe	155	4,178,956	3.71	3.49255	22.47
Lixian	140	4,933,150	2.84	2.37862	4.17
Huinxian	49	2,311,368	2.12	1.693	−3.85
Liangdang	6	476,876	1.26	0.78833	36.57
Linxia city	559	3,397,176	16.45	14.998	−6.74
Linxia county	335	3,649,890	9.18	7.48987	44.09
Kangle	140	2,702,683	5.18	5.01555	24.5
Yongjing	205	2,017,772	10.16	8.20293	23.8
Guanghe	99	2,688,469	3.68	4.49441	27.19
Hezheng	127	2,166,996	5.86	5.82397	7.5
Dongxiang	116	3,197,931	3.63	4.27671	−4.74
Jishishan	105	2,658,851	3.95	3.76095	12.8
Hezuo	79	1,093,849	7.22	8.80162	−0.84
Lintan	61	1,489,724	4.09	3.60823	13.29
Zhuoni	45	1,102,208	4.08	3.96479	17.12
Zhouqu	68	1,450,800	4.69	4.31253	2.9
Diebu	19	580,294	3.27	3.5862	13.43
Maqu	15	619,958	2.42	3.02005	9.68
Luqu	15	398,525	3.76	4.85625	6.8
Xiahe	41	962,433	4.26	4.05751	20.16

ASIR, age-standardized incidence rate.

AAPC, annual average percentage change.

#### 3.1.4 Temporal trends in CRC incidence in Gansu Province

**Figure 5:**
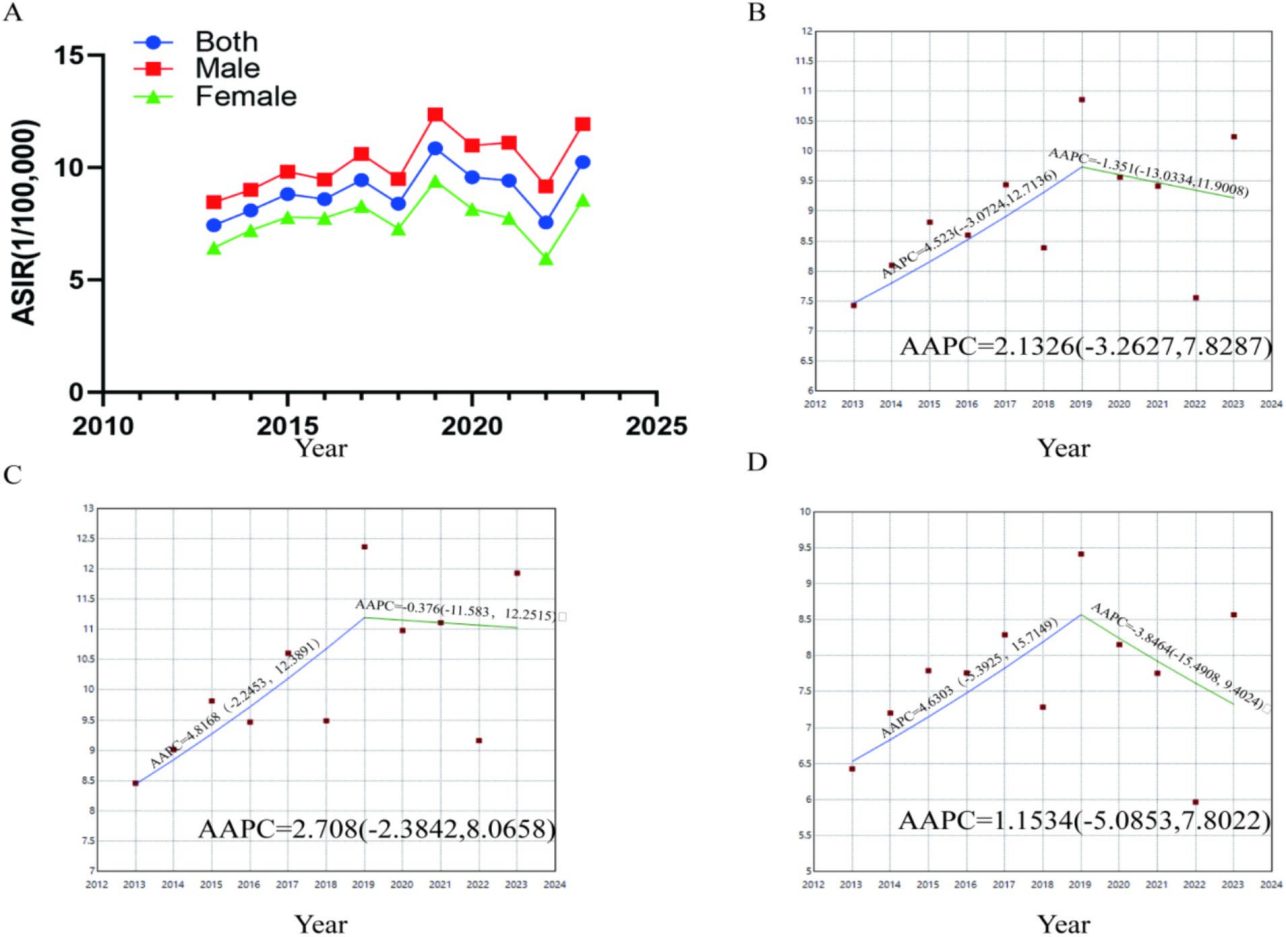
Temporal trends in age-standardized incidence rates (ASIR) of CRC in Gansu Province, 2013–2023. **(A)** Temporal changes in age-standardized incidence rates (ASIR). **(B)** Joinpoint regression analysis of ASIR among all patients. **(C)** Joinpoint regression analysis of ASIR among male patients. **(D)** Joinpoint regression analysis of ASIR among female patients.

As shown in Figure 5A and Supplementary Table 2, our analysis indicates a generally upward trend in the age-standardized incidence rate (ASIR) of CRC in Gansu Province from 2013 to 2023. JoinPoint regression identified two separate time segments within this timeframe (Figures 5B,C). As shown in Supplementary Table 1, that the overall average annual percent change (AAPC) was 2.13% (95% CI: −3.26 to 7.83, *p* = 0.45). For males, the AAPC was 2.71% (95% CI: −2.38 to 8.07, *p* = 0.30), while for females, it was 1.15% (95% CI: −5.09 to 7.80, *p* = 0.30). From 2013 to 2019, the overall annual percent change (APC) was 4.52% (95% CI: −3.07 to 12.71, *p* = 0.20). During this period, the APC for males was 4.82% (95% CI: −2.25 to 12.39, *p* = 0.15), and for females, it was 4.63% (95% CI: −5.39 to 15.71, *p* = 0.31). However, between 2019 and 2023, the incidence rate experienced a slight decrease, with an overall APC of −1.35% (95% CI: −13.03 to 11.90, *p* = 0.80). For males, the APC was −0.38% (95% CI: −11.58 to 12.25, *p* = 0.94), and for females, it was −3.85% (95% CI: −15.49 to 9.40, *p* = 0.49). Overall, the data indicate that the incidence rate has been increasing more rapidly among males than females during the study period.

### 3.2 Temporal and spatial epidemiological analysis of CRC in Gansu Province

#### 3.2.1 Assessment of spatial dependence in the dataset

**Figure 6:**
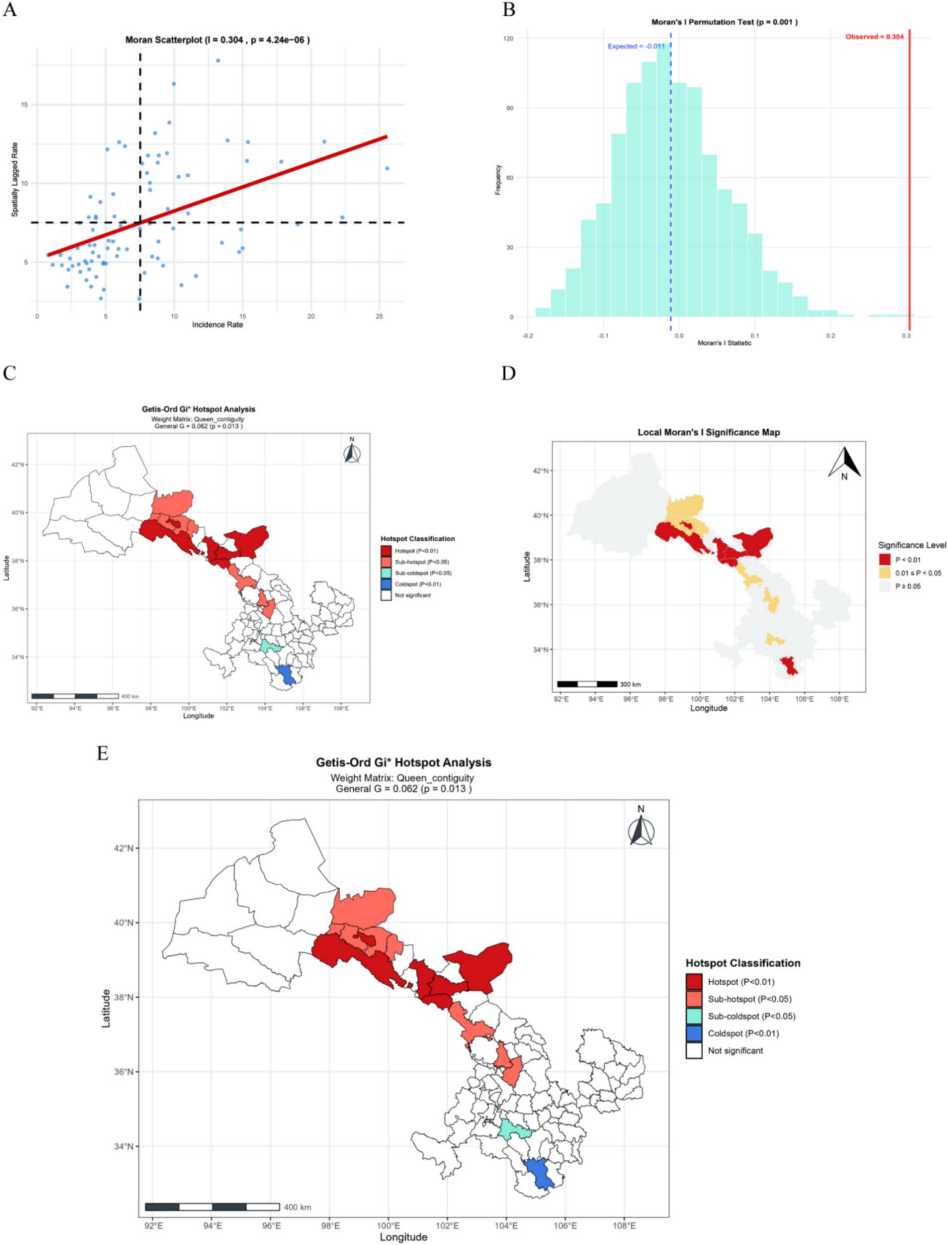
Spatial distribution analysis. **(A)** Scatter plot of Moran’s I for global spatial autocorrelation analysis. **(B)** Permutation test for global spatial autocorrelation analysis. **(C)** Cluster map of local spatial autocorrelation analysis. **(D)** Significance map of local spatial autocorrelation analysis. **(E)** Hot spot analysis of CRC incidence in Gansu Province.

We conducted both global and local spatial autocorrelation analyses to explore the clustering patterns of CRC incidence in Gansu Province. The results of the Global Moran’s *I* (Moran’s *I* = 0.304, *Z* = 4.4526, *p* < 0.001; see Figures 6A,B) revealed significant spatial clustering. To further investigate this spatial structure, we performed a hot spot analysis, which demonstrated a clear geographic pattern of disease distribution. The Global Getis-Ord General G statistic was 0.0545 (*p* = 0.0123), further confirming the presence of significant spatial clustering in the distribution of colorectal cancer incidence (As shown in Figure 6E). Central and northern regions of Gansu emerged as high-incidence core areas, whereas southern regions such as Min County, Wudu District, and Huixian County were identified as cold spots with comparatively low incidence rates. As shown in Figures 6C,D, the Local Moran’s I analysis identified several statistically significant high-high clusters, including Sunan Yugur Autonomous County, Jiayuguan City, Gaotai County, Linze County, Minqin County, Tianzhu Tibetan Autonomous County, Jinchuan District, Yongchang County, and Yuzhong County. Conversely, low-low clusters were mainly located in Min County and Wudu District. In addition, low-high clusters were observed in Jinta County and Gaolan County. Notably, no significant high-low clusters were detected across the province.

#### 3.2.2 SaTScan-based analysis of spatiotemporal aggregation

Using SaTScan v10.3, we conducted both purely spatial and retrospective space–time scan analyses of colorectal cancer incidence across 87 regions from 2013 to 2023, based on a discrete Poisson model. In addition, stratified spatial analyses were performed by age group (young, middle-aged, and older population) to explore age-specific spatial distribution patterns.

**Figure 7:**
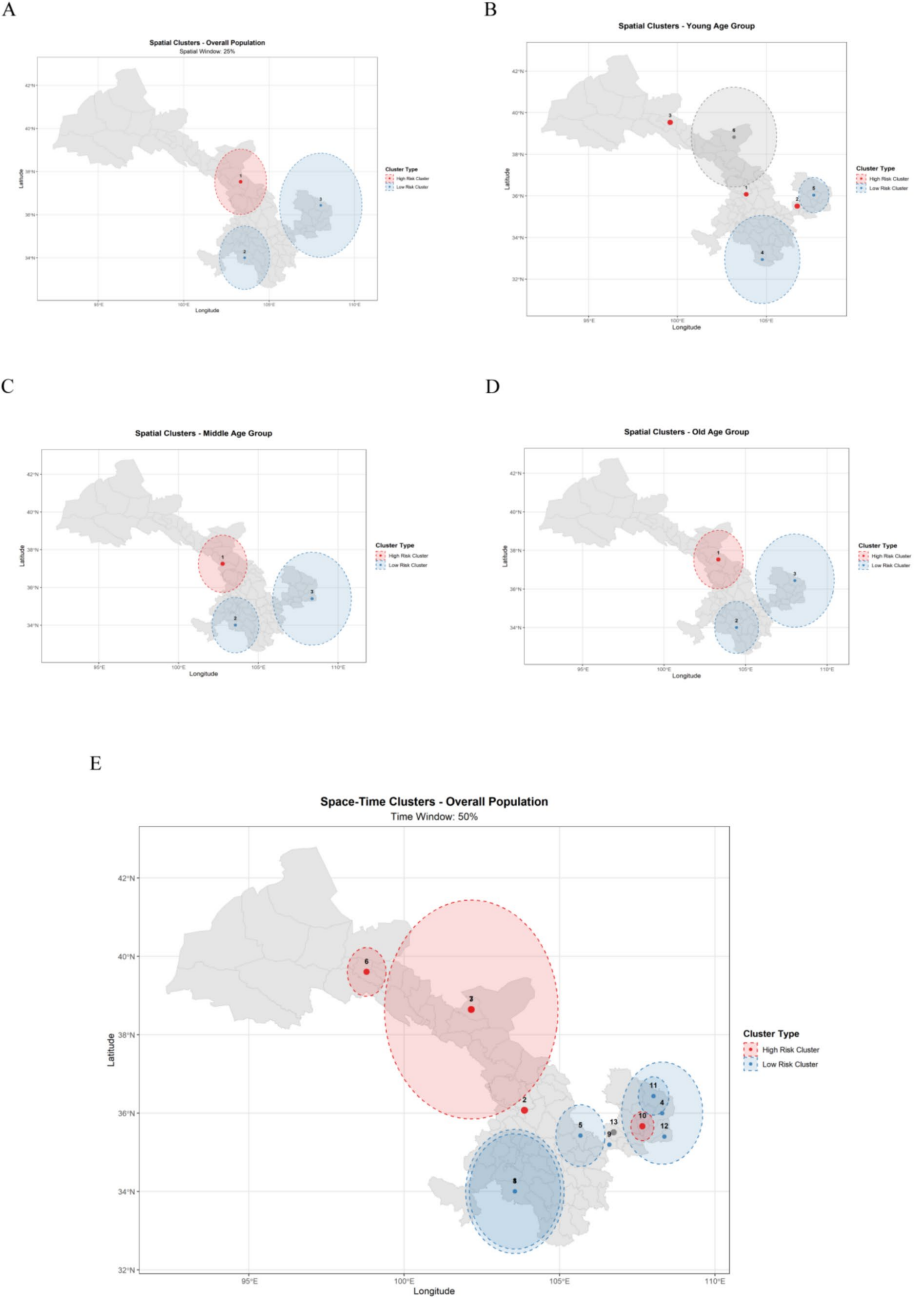
Space–time scan analysis of CRC in Gansu. **(A)** Pure spatial cluster map of CRC in Gansu Province. **(B)** Spatial scan statistic cluster map of colorectal cancer among young adults in Gansu Province. **(C)** Spatial scan statistic cluster map of colorectal cancer among middle adults in Gansu Province. **(D)** Spatial scan statistic cluster map of colorectal cancer among old adults in Gansu Province. **(E)** Space–time cluster map of CRC in Gansu Province.

In the overall spatial analysis (combining all age groups), three statistically significant clusters were identified (Figure 7A and Supplementary file 1).

The most prominent cluster (Cluster 1) represented a high-risk area centered at (37.5263° N, 103.3310° E) with a radius of 168.43 km, covering 16 regions. The observed number of cases (11,113) exceeded the expected count (6,514.56), yielding an observed-to-expected ratio (O/E) of 1.71, a relative risk (RR) of 2.18, and a log likelihood ratio (LLR) of 1881.57 (*p* < 0.001). The annual incidence rate in this cluster was 16.7 per 100,000, notably higher than the provincial average (9.8 per 100,000). Cluster 2, located in the southern region, was identified as a low-risk area (center: 34.0047° N, 103.5620° E) encompassing 19 regions, with 2,053 observed cases, substantially lower than expected (O/E = 0.43, RR = 0.38, LLR = 1160.72, *p* < 0.001). Cluster 3, a second low-risk area situated in the eastern region (center: 36.4380° N, 108.0210° E), showed an O/E ratio of 0.67 (RR = 0.62, LLR = 461.24, *p* < 0.001).

The space–time scan analysis revealed clear spatiotemporal dynamics of colorectal cancer risk (Figure 7E and Supplementary file 5). The most significant space–time cluster (Cluster 1) corresponded to a persistent low-risk zone in the southern area during 2013–2017, covering 23 locations (LLR = 1493.72, *p* < 0.001), where the observed cases accounted for only 20% of the expected number (O/E = 0.20). Concurrently, a localized high-risk cluster (Cluster 2) was detected in Chengguan District (central region) during 2013–2017, where the observed incidence was 3.36 times higher than expected (RR = 3.56, LLR = 1122.90, *p* < 0.001). Another notable high-risk cluster (Cluster 3) emerged in the northwestern region during 2019–2023, encompassing 21 locations, with an O/E ratio of 1.87 (RR = 2.08, LLR = 968.42, *p* < 0.001). Several secondary clusters were also identified, including two eastern low-risk zones (Clusters 4 and 5) during 2013–2017, and several localized high-risk foci (Clusters 6 and 7).

Distinct age-specific clustering patterns were observed (Figures 7B–D and Supplementary files 2–4). For the young group, the overall incidence rate was lowest (0.6 per 100,000) but exhibited extremely localized high-risk foci. Three major high-risk clusters were detected in Chengguan District (O/E = 3.41), location 620,802 (O/E = 5.21), and location 620,724 (O/E = 10.00, RR = 10.41), followed by two broad low-risk regions (Clusters 4 and 5). This pattern suggests that the disease risk among younger individuals is highly geographically concentrated. The middle-aged group showed a spatial pattern similar to the total population, but with attenuated relative risks—one high-risk cluster (Cluster 1, RR = 1.97) and two low-risk clusters (Clusters 2 and 3; O/E = 0.50 and 0.67) were detected. The older population group, with the highest incidence (39.4 per 100,000), exhibited a clustering structure similar to that of the middle-aged group but with greater spatial contrast—the high-risk cluster (Cluster 1, RR = 2.26) and low-risk cluster (Cluster 2, RR = 0.43) both demonstrated stronger relative differences, indicating more pronounced geographic disparities in this age group.

### 3.3 Environmental factors and colorectal cancer incidence in Gansu Province: a correlation analysis

#### 3.3.1 Geodetector analysis of contributing factors

This study systematically investigated the spatial relationships between environmental factors and colorectal cancer (CRC) incidence across county-level divisions in Gansu Province using Geodetector modeling. By integrating multi-source environmental data with mean values calculated for the 2003–2013 period, We found that almost all of the analyzed environmental variables demonstrated a statistically significant association with the incidence of CRC (*p* < 0.05, Table 2).

**Table 2:** Geodetector factor detector results for CRC.

Variable	*q*_value	*p*_value	Permutation_*p*_value
CH4	0.295	*P* < 0.001	0.02
CO2	0.378	*P* < 0.001	0.02
GHG	0.345	*P* < 0.001	0.02
NDVI	0.323	*P* < 0.001	0.02
Nox	0.223	0.0015	0.02
O3	0.238	*P* < 0.001	0.02
OC	0.141	0.026	0.1
PM10	0.277	0.0002	0.02
Precipitation levels	0.343	*P* < 0.001	0.02
SO2	0.192	0.0047	0.02
Tem	0.172	0.0104	0.02
CO	0.122	0.048	0.04
Slpoe	0.258	*P* < 0.001	0.02
Humidity	0.269	*P* < 0.001	0.02
GDPper	0.346	*P* < 0.001	0.02
Number of hospital beds	0.177	0.0084	0.02
Value of the first industry	0.071	0.2238	0.28
Value of the second industry	0.319	*P* < 0.001	0.02
Value of the third industry	0.085	0.1151	0.08
GDP	0.405	*P* < 0.001	0.02

The *q*-statistic analysis revealed that each environmental factor exerted significant influence on disease incidence (Almost all *q*-values ≥ 0.1). Seven key drivers emerged with particularly strong explanatory power for the spatial heterogeneity of CRC distribution (*q*-values > 0.3): GDP, CO2, GDPper, GHG, Precipitation levels, NDVI and Value of the Second industry. The remaining factors demonstrated relatively weaker associations (*q*-values < 0.3). Of the 20 variables analyzed, 17 demonstrated statistically significant associations based on permutation tests (Permutation *p*-value < 0.05). Notably, several key environmental and socioeconomic factors—including CO₂, GDP, GDP per capita, rainfall, and greenhouse gases—showed particularly strong associations, each with q-values below 0.4 and permutation *p*-values of 0.02. It is worth noting that the variable OC, which showed marginal significance in the parametric test (*p* = 0.026), was not statistically significant under the permutation test framework (Permutation *p*-value = 0.1). This suggests that the observed effect of OC may be sensitive to distributional assumptions and lacks robustness. Furthermore, two variables—the value of the primary industry and the value of the tertiary industry—failed to show statistical significance in both testing approaches, with permutation *p*-values of 0.28 and 0.08, respectively. This indicates that their influence on the target factor may be negligible or non-existent.

#### 3.3.2 Geodetector interaction detector results

**Figure 8:**
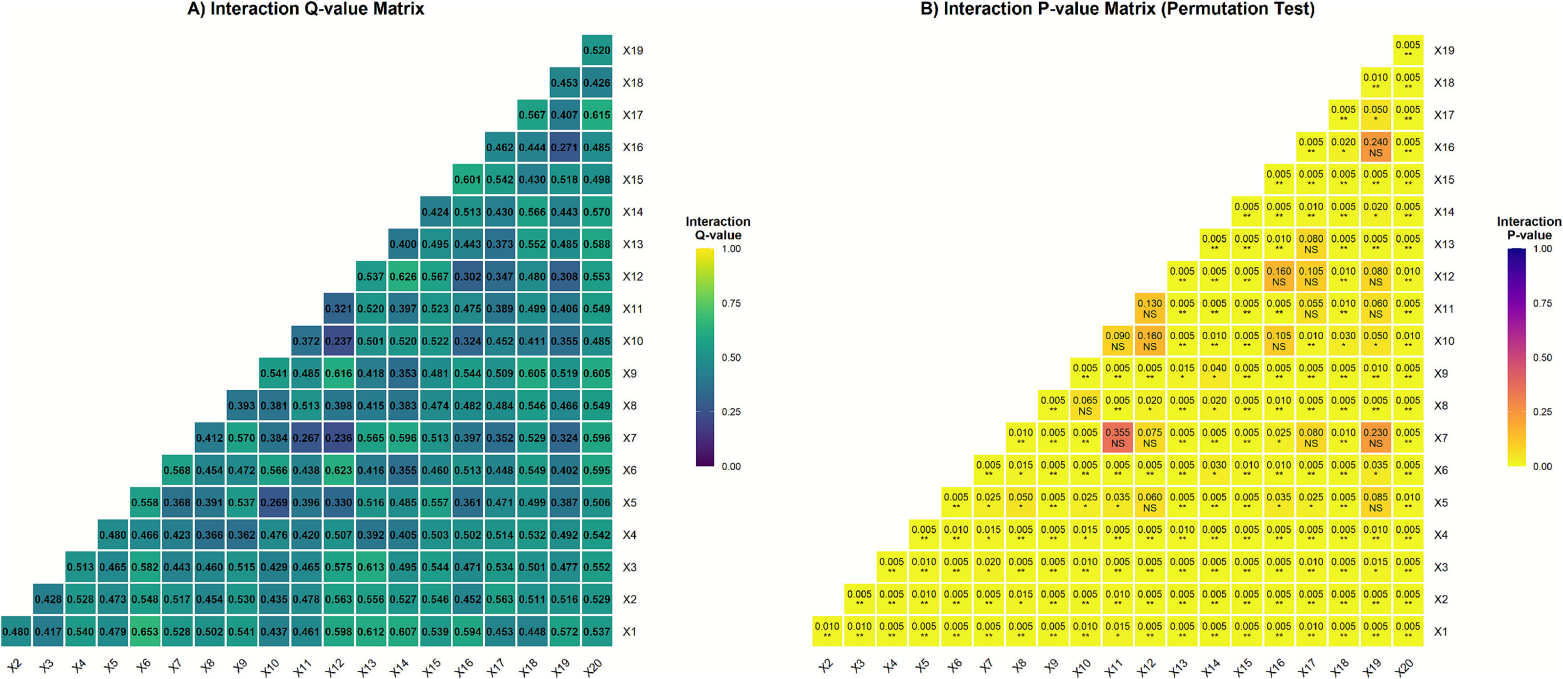
Interactive effects of risk factors on the incidence of CRC. **(A)** The *q*-statistic for measuring interaction, with error bars indicating 95% confidence intervals. **(B)** The statistical significance of interactions assessed by permutation *p*-values. (X1, CH_4_; X2, CO_2_; X3, GHG; X4, NDVI; X5, NO_x_; X6, O_3_; X7, OC; X8, PM10; X9, Precipitation; X10, SO_2_; X11, Tem; X12, CO; X13, Slpoe, X14, Humidity. X15, GDPper; X16, Number of hospital beds; X17, Value of the first industry, X18, Value of the second industry; X19, Value of the third industry; X20, GDP).

Based on the interaction detection results of the Geodetector, the influence of various environmental factors on the spatial differentiation of total_ASIR exhibited a pronounced enhancement effect through interaction (Figure 8). The *Q*-value matrix analysis indicated that the interactions among atmospheric pollutants were particularly notable. Among them, the interaction between CH₄ (X1) and O₃ (X6) was the strongest (*Q* = 0.653), followed by the interaction between CO (X12) and O₃ (X6) (*Q* = 0.623). These findings suggest that different air pollutants may exert synergistic health effects through complex atmospheric chemical processes.

The interactions between pollutants and topographic or meteorological factors were also significant (Figure 8A). The strong interaction between CH₄ (X1) and slope (X13) (*Q* = 0.612) implies that terrain conditions play a crucial role in influencing the dispersion and accumulation of pollutants. Similarly, the interaction between humidity (X14) and CO (X12) (*Q* = 0.626) indicates that meteorological conditions regulate pollutant transformation and deposition, thereby modulating associated health risks. These results confirm that nonlinear coupling among environmental factors is a key mechanism shaping the spatial pattern of health risks.

Regarding socioeconomic and healthcare factors, the significant interaction between GDP (X20) and precipitation (X9) (*Q* = 0.605) highlights the combined effects of economic development and climatic conditions. Meanwhile, the relatively strong interaction between per capita GDP (X15) and number of hospitals (X16) (*Q* = 0.601) reflects the synergistic influence of economic level and healthcare resources. In contrast, the interactions between healthcare resources and major pollutants were comparatively weaker, suggesting that, at the current spatial scale, environmental drivers may outweigh the moderating effects of medical resources.

Permutation tests showed that all key interactions passed the significance threshold (*p* < 0.01), confirming the robustness of these findings (Figure 8B). Overall, this study underscores the importance of integrating multiple environmental and socioeconomic interactions—including pollutants, meteorological, topographic, and economic factors—when assessing environmental health risks.

#### 3.3.3 Multivariate spatial analysis

To identify the model that best characterizes the spatial structure of the data, we systematically compared the Ordinary Least Squares (OLS) model with a series of spatial econometric models (see Table 1). The comparison results indicated that the Spatial Error Model (SEM) performed best, yielding the lowest values of both AIC (451.17) and BIC (473.37) among all candidate models. Moreover, the spatial error coefficient of SEM was highly significant (*λ* = 0.4004, *p* < 0.001), and its goodness of fit (R^2^ = 0.6653) exceeded that of the OLS baseline model. These statistical results consistently suggest that the spatial dependence within the study system primarily manifests through spatial autocorrelation in the error term. Therefore, the SEM was selected as the final model for subsequent analysis and interpretation.

Based on the final SEM specification, the estimated coefficients and significance levels of the explanatory variables are summarized in Table 2. After controlling for spatial effects, GDP emerged as the only statistically significant predictor (*p* < 0.001), with a coefficient of 3.166, indicating a strong positive influence of economic development on the dependent variable. In contrast, other environmental factors—including CH₄, O₃, CO, and humidity—did not exhibit statistically significant effects (*p* > 0.05). Additionally, although the slope variable narrowly missed the conventional significance threshold (*p* = 0.058), its negative coefficient (−1.232) implies a potential downward trend that may warrant further investigation.

## 4 Discussion

The global incidence of colorectal cancer (CRC) is steadily increasing, with significant rises reported in China, Vietnam, and several other countries (15, 16). And CRC incidence remains higher and is increasing more rapidly in males, both globally and in China (17). Similarly, epidemiological studies indicate a consistent increase in CRC cases in Gansu Province, with a significantly higher incidence in males compared to females. The majority of cases are found in individuals aged 50 and older. The gender disparity may be linked to higher estrogen levels in females, which have anti-proliferative and apoptotic effects through the modulation of estrogen receptors (ERα and ERβ), thereby helping to inhibit CRC development (18, 19). The higher prevalence of smoking and alcohol consumption among males, both established risk factors for colorectal cancer, further explains the observed gender disparity in disease incidence (20, 21). Understanding the overall epidemiological trends of colorectal cancer, as well as gender-specific variations, enables more targeted prevention strategies for different population groups to reduce disease incidence.

Analysis of colorectal cancer incidence patterns across various counties and districts in Gansu Province during 2013–2023 reveals significant spatiotemporal variations in disease distribution. This research employed spatial autocorrelation and temporal scanning methods to analyze the spatial and temporal patterns of CRC incidence in Gansu Province. The global Moran’s I index was calculated at 0.304 (*p* < 0.001), indicating significant spatial clustering. Local analysis identified a high-high aggregation area in the north-central region, particularly in Sunan County and Jiayuguan City (all *p* values <0.05), while the southern region, including Min County and Wudu District, showed a low-low aggregation area. Additionally, given the unique low-high aggregation areas in Jinta and Gaolan counties, it is recommended to verify data quality and conduct specific epidemiological studies. The spatial and temporal scanning results confirmed these patterns, revealing that the central high-risk zone’s risk increased from 2019 to 2023 (RR = 2.08), while the southern low-risk zone experienced a slight increase in risk during the later period (2019–2022) (RR = 0.63). Notably, Chengguan District in Lanzhou City had an exceptionally high risk from 2013 to 2017 (RR = 3.56), with an average annual incidence rate of 32.9 per 100,000, significantly above the provincial average of 9.8 per 100,000. These geographical and spatiotemporal variations likely arise from the complex interplay of multiple factors, including the province’s diverse topography, regional disparities in air quality levels, and significant climatic differences across different zones (22, 23). Gansu Province exhibits pronounced geographic and spatiotemporal heterogeneity across its counties, attributable to its elongated topographic diversity. The findings collectively emphasize that CRC risk in Gansu Province is shaped by multifaceted environmental exposures that vary substantially across its distinct geographical regions, necessitating tailored prevention approaches that account for these local environmental characteristics (24).

Our geodetector analysis revealed 18 environmental factors showing significant associations with colorectal cancer incidence in Gansu Province, with particularly strong correlations observed for humidity, vegetation index (NDVI), and airborne pollutants including greenhouse gases, PM10, and organic carbon. The results of the permutation test provide a scientific basis for identifying priority targets for intervention. Factors such as organic carbon (OC), which did not pass the robustness test, may have limited public health relevance. In contrast, the core driving factors that withstood the test—such as CO₂ and GDP—should be regarded as key focuses in developing precise environmental health risk prevention and control strategies. Importantly, these environmental factors exhibited synergistic interactions, where their combined effects on CRC risk exceeded the sum of their individual impacts, highlighting the critical need for comprehensive environmental health strategies in CRC prevention programs.

Using the Geodetector approach, this study systematically analyzed the influencing factors and interactions underlying the spatial distribution of colorectal cancer (CRC) (25). The findings revealed a significant positive correlation between the level of economic development and CRC incidence, consistent with numerous national and international studies (26). Notably, this association exhibits a dual nature: on one hand, economically developed regions tend to report higher detection rates due to better healthcare infrastructure and screening capacity; on the other hand, changes in dietary patterns—such as increased consumption of high-fat foods and processed meats—along with industrialization-induced environmental pollution, jointly contribute to an elevated risk of CRC (27–29). The study further identified a strong spatial association between secondary industry output and CRC incidence. This linkage may operate through two primary pathways. Firstly, the expansion of the food processing industry increases exposure to harmful substances such as nitrites in processed meats, consistent with findings from the World Cancer Research Fund (30, 31). Secondly, industrial pollution may disrupt the gut microenvironment, thereby facilitating CRC development (32, 33). In addition, the analysis highlighted that the distribution of medical resources plays a critical role in disease surveillance capacity. Regions with abundant healthcare resources are more capable of early diagnosis, but this may also introduce spatial bias in incidence data (33, 34). This underscores the need to account for inequalities in healthcare access when comparing disease burdens across regions. Overall, these findings provide valuable guidance for region-specific CRC prevention and control strategies. While promoting economic development, efforts should also focus on strengthening environmental pollution control, encouraging healthy dietary habits, and fostering intersectoral collaboration to achieve a balanced relationship between economic growth and public health.

Epidemiological analysis reveals a positive association between colorectal cancer incidence and ambient air pollution levels, with significantly higher case clustering observed in regions with elevated pollution exposure (31). This trend may be linked to various factors, including air pollution and food processing. A 2007 report from the World Cancer Research Fund (WCRF) identified processed meat consumption as a risk factor for CRC due to its high nitrite and nitrate content (30). Additionally, the use of synthetic food colorings, monosodium glutamate (MSG), and industrial trans fats in food production contributes to the increased risk of developing CRC (32). Industrial development has been associated with environmental pollution, and numerous epidemiological studies have suggested a link between exposure to air pollution and CRC (35). Research has shown that outdoor particulate air pollution can contribute to the onset of gastrointestinal cancers in adults (36). Furthermore, greenhouse gases produced by industry, such as CH4, CO2, GHG and CO, have been found to adversely affect CRC cells (37). Studies have also indicated that sulfur dioxide (SO2) from industrial waste significantly impacts colorectal development (38). Chronic exposure to fine particulate matter, like PM10, has been shown to cause dyslipidemia, which is a notable risk factor for CRC (39, 40). Certain studies have found that air pollutants, particularly organic carbon (OC), can lead to dyslipidemia, potentially resulting in cancer development (41). Increased levels of ozone (O3) from vehicle emissions and industrial pollution indirectly affect human health and contribute to cancer risk (42). Evidence indicates that the influence of industrial development on CRC differs across regions (43). In rapidly industrializing areas, it is crucial to improve the environment to reduce associated risks. It is suggested that changes in geography and ecology could contribute to the development of CRC and it can be argued that the higher incidence of CRC in warmer areas is linked to a greater consumption of processed foods, red meat, and high-fat diets, such as barbecued items. Futhermore,this dietary trend is marked by a lack of fruits and vegetables, which are known to increase the risk of CRC (44, 45). However, more research is needed to confirm this observation. Additionally, it is proposed that differences in precipitation among various regions may correlate with a higher risk of developing CRC. Drier areas tend to consume more preserved foods, which contain high levels of nitrates, nitrites, N-dimethyldinitrosamines, and N-ethylnitrosamines—substances that are associated with CRC risk—compared to regions with more rainfall (46, 47). Studies have shown that areas with higher Normalized Difference Vegetation Index (NDVI) levels have lower incidence rates of CRC, likely due to their better air purification capabilities and reduced UV exposure, leading to a healthier environmental profile (48). Experimental studies hypothesize that thermal stress from climate variability could impair intestinal microbial ecology, creating a permissive microenvironment for colorectal cancer development (9, 49, 50). Emerging evidence suggests that environmental humidity and terrain slope may influence the incidence of colorectal cancer (CRC), though no direct causal relationship has been established in existing literature. Studies indicate that prolonged exposure to high-humidity environments may disrupt gut microbiota homeostasis and promote bacterial translocation, potentially triggering chronic inflammation—a known risk factor for carcinogenesis. However, further research is needed to clarify the mechanistic links between these environmental factors and CRC development (51, 52). Additionally, terrain slope may indirectly contribute to colorectal cancer (CRC) risk by modulating local climatic conditions, such as temperature and precipitation patterns, which could subsequently affect dietary habits, microbial exposure, or inflammatory pathways. However, the specific mechanisms remain speculative and warrant further investigation (53, 54).

This study developed a set of spatial multivariate regression models to systematically compare the performance of traditional Ordinary Least Squares (OLS) and spatial econometric models, demonstrating the necessity of accounting for spatial dependence when analyzing geographic data. The comparison results revealed that the Spatial Error Model (SEM) provided the best overall fit, indicating a significant spatial autocorrelation within the study system. After controlling for this spatial effect, the multivariate regression results changed markedly: most local environmental variables, such as CH₄, O₃, and CO, lost their statistical significance, whereas GDP remained a robust and highly significant predictor. This pattern suggests that spatial dependence may share collinearity with certain local environmental factors, and that the SEM, by absorbing unobserved spatial components, offers more reliable parameter estimates. Overall, the findings emphasize the dominant role of macroeconomic drivers (GDP) in shaping the studied phenomenon and confirm that extending the model specification from OLS to a spatial regression framework is crucial for ensuring the robustness of multivariate analysis, enabling the identification of truly stable and influential driving factors among many correlated variables.

This research is the first thorough examination of CRC epidemiology, high-risk regions, and environmental factors across all 87 counties in Gansu Province. Unlike earlier studies that focused on specific cancer registry sites, our analysis across the entire province offers more accurate and objective estimates of CRC incidence at the county level. However, there are several limitations to this study. Firstly, we could not include several known risk factors for CRC (such as smoking, obesity, alcohol use, and dietary habits) due to a lack of data at the county level. Secondly, there may be slight biases in the incidence estimates due to population movement and the exclusion of cases with incomplete residential data. Another limitation of this study is that, as illustrated in Figure 5A, the incidence rate showed a marked decline between 2020 and 2023. This observed decrease is unlikely to reflect genuine epidemiological changes in cancer incidence and is instead primarily attributable to the global COVID-19 pandemic. During this period, constraints on healthcare resources, disruptions or delays in cancer screening and diagnostic services, and alterations in patient health-seeking behaviors may have collectively contributed to the underreporting and delayed registration of new cases. Furthermore, the operation of cancer surveillance systems themselves may have been compromised, affecting data completeness and accuracy. Consequently, the apparent decline in incidence during this timeframe should be interpreted with caution, as it likely represents a data artifact induced by an extraordinary public health crisis rather than a true reversal of long-term trends. Lastly, although we analyzed 20 environmental factors, our study was not exhaustive, and further research is needed to explore the underlying mechanisms and validate these findings to gain a better understanding of the observed relationships.

## 5 Conclusion

Our research uncovered notable differences in the incidence of CRC throughout Gansu Province, showing unique epidemiological trends and areas with elevated risk. The geodetector analysis indicated that ecological factors and air pollution are major influences on the distribution of CRC. These results emphasize the need for focused interventions that tackle these adjustable risk factors to lessen the impact of CRC in the area.

## Data Availability

The original contributions presented in the study are included in the article/Supplementary material, further inquiries can be directed to the corresponding author/s.
