# Supplementary material for: Integrating geospatial and environmental factors in colorectal cancer epidemiology: a regional study
Source: Front Public Health. 2026 Jan 15;13:1699870. doi: 10.3389/fpubh.2025.1699870 (PMC12852315; doi:10.3389/fpubh.2025.1699870)
Supplement: Supplementary file 2 [file Table_1.docx]

Supplementary Table 1. Environmental Variables Statistics

| Variable | Mean | SD | Min | Max | Variance |
| --- | --- | --- | --- | --- | --- |
| CH4（metric tons per year (t/year)） | 8453.27 | 15330.67 | 1030.92 | 109451.41 | 235029576 |
| CO2（Metric tons per year (t/year)） | 1641035.25 | 3037676.46 | 40155.88 | 15358065.91 | 9227478276161.77 |
| GHG(Metric tons CO?-equivalent per year (tCO?e/year)) | 1930601.96 | 2995254.69 | 177654.34 | 14607179.25 | 8971550641744.75 |
| NDVI | 0.52 | 0.22 | 0.07 | 0.86 | 0.05 |
| Nox（Metric tons per year (t/year)） | 351.49 | 698.44 | 2.97 | 4367.77 | 487819.29 |
| O3Parts Per Billion (ppb) | 85.52 | 5.99 | 76.84 | 101.98 | 35.84 |
| OC（μg/m3） | 47.45 | 75.66 | 0.17 | 480.25 | 5723.85 |
| PM10(μg/m3) | 107.73 | 22.01 | 66.3 | 181.59 | 484.33 |
| Precipitation(Millimeters (mm) | 461.74 | 194.66 | 34.9 | 753.09 | 37891.19 |
| SO2 | 448.69 | 1028.81 | 0.36 | 5543.08 | 1058440.48 |
| Tem(℃） | 7.04 | 2.87 | -2.19 | 11.77 | 8.24 |
| CO(Parts Per Billion (ppb) | 2504.62 | 9562.16 | 4.68 | 86537.53 | 91434877.33 |
| Slpoe(Percent (%)) | 14.8 | 6.97 | 1.61 | 30.83 | 48.58 |
| Humidity(Percent (%)) | 59.44 | 8.41 | 35.08 | 73.03 | 70.76 |
| GDPper(Chinese Yuan per person per year (CNY/capita/year)) | 15587.71 | 18257.22 | 2469.73 | 114269.36 | 333325909.1 |
| Number of Hospital Beds(Unit) | 699.84 | 507.48 | 70.73 | 3517.36 | 257536.98 |
| Primary Industry Output Value（10,000 Chinese Yuan (CNY)） | 54021.55 | 44634.13 | 2088.15 | 316742.18 | 1992205144 |
| Second Industry Output Value（10,000 Chinese Yuan (CNY)） | 173903.96 | 262797.02 | 4904.36 | 1500751.54 | 69062272296 |
| Third Industry Output Value（10,000 Chinese Yuan (CNY)） | 143449.78 | 291471.69 | 12110.82 | 2564997.36 | 84955748528 |
| GDP(Chinese Yuan per person per year (CNY/capita/year)) | 386608.9 | 501369.03 | 31427.91 | 3305386.35 | 251370901772.45 |
