# Supplementary material for: Integrating geospatial and environmental factors in colorectal cancer epidemiology: a regional study
Source: Front Public Health. 2026 Jan 15;13:1699870. doi: 10.3389/fpubh.2025.1699870 (PMC12852315; doi:10.3389/fpubh.2025.1699870)
Supplement: Supplementary file 3 [file Table_2.docx]

Supplemental Table 2.Specific values of Joinpoint regression for age-standardized incidence rates (ASIR) of colorectal cancer patients.

| Sex | Joinpoint Model | Start Obs | End Obs | AAPC | AAPC C.I. Low | AAPC C.I. High | Statistically Significant (0=No 1=Yes) | Test Statistic | P-Value |
| --- | --- | --- | --- | --- | --- | --- | --- | --- | --- |
| Both | 1 | 2013 | 2023 | 2.1326 | -3.2627 | 7.8287 | 0 | 0.762 | 0.446035 |
| Manle | 1 | 2013 | 2023 | 2.708 | -2.3842 | 8.0658 | 0 | 1.0299 | 0.303071 |
| Female | 1 | 2013 | 2023 | 1.1534 | -5.0853 | 7.8022 | 0 | 0.3531 | 0.724021 |
