# Supplementary material for: Integrating geospatial and environmental factors in colorectal cancer epidemiology: a regional study
Source: Front Public Health. 2026 Jan 15;13:1699870. doi: 10.3389/fpubh.2025.1699870 (PMC12852315; doi:10.3389/fpubh.2025.1699870)
Supplement: Supplementary file 5 [file Table_4.docx]

_____________________________

SaTScan v10.3

_____________________________

Program run on: Fri Oct 24 21:33:35 2025

Purely Spatial analysis

scanning for clusters with high or low rates

using the Discrete Poisson model.

_______________________________________________________________________________________________

SUMMARY OF DATA

Study period.......................: 2013/1/1 to 2023/12/31

Number of locations................: 87

Population, averaged over time.....: 11794103

Total number of cases..............: 736

Annual cases / 100000..............: 0.6

_______________________________________________________________________________________________

CLUSTERS DETECTED

1.Location IDs included.: 620102

Coordinates / radius..: (36.074300 N, 103.874000 E) / 0 km

Span..................: 0 km

Population............: 616122

Number of cases.......: 131

Expected cases........: 38.45

Annual cases / 100000.: 1.9

Observed / expected...: 3.41

Relative risk.........: 3.93

Log likelihood ratio..: 74.469522

P-value...............: 0.001

2.Location IDs included.: 620802

Coordinates / radius..: (35.510900 N, 106.738000 E) / 0 km

Span..................: 0 km

Population............: 236633

Number of cases.......: 77

Expected cases........: 14.77

Annual cases / 100000.: 3.0

Observed / expected...: 5.21

Relative risk.........: 5.71

Log likelihood ratio..: 67.691835

P-value...............: 0.001

3.Location IDs included.: 620724

Coordinates / radius..: (39.538800 N, 99.592800 E) / 0 km

Span..................: 0 km

Population............: 51273

Number of cases.......: 32

Expected cases........: 3.20

Annual cases / 100000.: 5.7

Observed / expected...: 10.00

Relative risk.........: 10.41

Log likelihood ratio..: 45.459601

P-value...............: 0.001

4.Location IDs included.: 621222, 621202, 623023, 621224, 621225, 621221, 621223, 621226,

621227, 623024, 621126, 620502, 621228, 620524, 621125, 620503,

620523, 623022, 623021, 620521

Coordinates / radius..: (32.942900 N, 104.775000 E) / 235.39 km

Span..................: 286.52 km

Population............: 2886506

Number of cases.......: 88

Expected cases........: 180.13

Annual cases / 100000.: 0.3

Observed / expected...: 0.49

Relative risk.........: 0.42

Log likelihood ratio..: 36.336567

P-value...............: 0.001

5.Location IDs included.: 621021, 621002, 621027, 621023, 621024, 621026, 620821, 621022,

621025

Coordinates / radius..: (36.040600 N, 107.672000 E) / 94.38 km

Span..................: 177.81 km

Population............: 1179197

Number of cases.......: 16

Expected cases........: 73.59

Annual cases / 100000.: 0.1

Observed / expected...: 0.22

Relative risk.........: 0.20

Log likelihood ratio..: 35.606300

P-value...............: 0.001

6.Location IDs included.: 620621, 620302, 620602, 620321, 620622, 620725, 620623, 620423,

620722, 620702, 620121, 620723, 620402

Coordinates / radius..: (38.829500 N, 103.184000 E) / 266.83 km

Span..................: 466.55 km

Population............: 1716934

Number of cases.......: 144

Expected cases........: 107.14

Annual cases / 100000.: 0.8

Observed / expected...: 1.34

Relative risk.........: 1.43

Log likelihood ratio..: 6.818000

P-value...............: 0.093

_______________________________________________________________________________________________

ADDITIONAL RESULTS FILES

Cluster Information : D:\colon cancer\time and space\young\high-low.col.txt

Cluster Information : D:\colon cancer\time and space\young\high-low.col.dbf

_______________________________________________________________________________________________

PARAMETER SETTINGS

Input

-----

Case File : D:\colon cancer\time and space\young\CAS.csv

Population File : D:\colon cancer\time and space\young\pop.csv

Time Precision : Year

Start Date : 2013/1/1

End Date : 2023/12/31

Coordinates File : D:\colon cancer\time and space\young\geo.csv

Coordinates : Latitude/Longitude

Analysis

--------

Type of Analysis : Purely Spatial

Probability Model : Discrete Poisson

Scan For Areas With : High or Low Rates

Output

------

Main Results File : D:\colon cancer\time and space\young\high-low.txt

HTML file for Google Map : No

KML file for Google Earth : No

Shapefile for GIS software : No

HTML file for Cartesian map : No

Cluster Information : Yes (ASCII), Yes (dBase)

Stratified Cluster Information : No (ASCII), No (dBase)

Location Information : No (ASCII), No (dBase)

Risk Estimates for Each Location : No (ASCII), No (dBase)

Simulated Log Likelihood Ratios : No (ASCII), No (dBase)

Data Checking

-------------

Temporal Data Check : Check to ensure that all cases and controls are within the specified temporal study period.

Geographical Data Check : Check to ensure that all observations (cases, controls and populations) are within the specified geographical area.

Spatial Neighbors

-----------------

Specify neighbors through a non-Euclidean neighbors file : No

Specify a meta location file : No

Observations with Multiple Locations : One location per observation.

Locations Network

-----------------

Use Locations Network File : No

Spatial Window

--------------

Maximum Spatial Cluster Size : 25 percent of population at risk

Window Shape : Circular

Isotonic Scan : No

Cluster Restrictions

--------------------

Minimum Cases in Cluster for High Rates : 2

Restrict High Rate Clusters : No

Restrict Low Rate Clusters : No

Space And Time Adjustments

--------------------------

Adjust for Known Relative Risks : No

Inference

---------

P-Value Reporting : Default Combination

Number of Replications : 999

Adjusting for More Likely Clusters : No

Drilldown

---------

Same Design as Main Analysis : No

Miscellaneous Analysis

----------------------

Report Oliveira's F : No

Spatial Output

--------------

Report Hierarchical Clusters : Yes

Criteria for Reporting Secondary Clusters : No Geographical Overlap

Report Gini Optimized Cluster Collection : No

Restrict Reporting to Smaller Clusters : No

Other Output

------------

Report Critical Values : No

Report Monte Carlo Rank : No

Print ASCII Column Headers : No

User Defined Title :

Notifications

-------------

Always Send Email : No

Send Email with Results Meeting Cutoff : No

Run Options

-----------

Processor Usage : All Available Processors

Suppress Warnings : No

Logging Analysis : No

_______________________________________________________________________________________________

RUN INFORMATION

Program completed : Fri Oct 24 21:33:36 2025

Total Running Time : 1 second

Processor Usage : 16 processors
