# Supplementary material for: Integrating geospatial and environmental factors in colorectal cancer epidemiology: a regional study
Source: Front Public Health. 2026 Jan 15;13:1699870. doi: 10.3389/fpubh.2025.1699870 (PMC12852315; doi:10.3389/fpubh.2025.1699870)
Supplement: Supplementary file 6 [file Table_5.docx]

SaTScan v10.3

_____________________________

Program run on: Fri Oct 24 21:23:02 2025

Purely Spatial analysis

scanning for clusters with high or low rates

using the Discrete Poisson model.

_______________________________________________________________________________________________

SUMMARY OF DATA

Study period.......................: 2013/1/1 to 2023/12/31

Number of locations................: 87

Population, averaged over time.....: 9911673

Total number of cases..............: 10091

Annual cases / 100000..............: 9.3

_______________________________________________________________________________________________

CLUSTERS DETECTED

1.Location IDs included.: 620623, 620622, 620602, 620121, 620111, 620423, 620321, 620122,

620104, 622923, 620402, 620105, 620302, 620102, 620103

Coordinates / radius..: (37.250400 N, 102.763000 E) / 168.56 km

Span..................: 330.01 km

Population............: 2452082

Number of cases.......: 3968

Expected cases........: 2496.45

Annual cases / 100000.: 14.7

Observed / expected...: 1.59

Relative risk.........: 1.97

Log likelihood ratio..: 519.980493

P-value...............: 0.001

2.Location IDs included.: 623024, 623022, 621126, 623021, 621223, 623023, 621125, 623026,

623001, 621123, 622922, 621226, 620524, 622925, 623027, 621122,

621222, 622924, 621225

Coordinates / radius..: (34.004700 N, 103.562000 E) / 163.18 km

Span..................: 311.91 km

Population............: 1637322

Number of cases.......: 840

Expected cases........: 1666.94

Annual cases / 100000.: 4.7

Observed / expected...: 0.50

Relative risk.........: 0.46

Log likelihood ratio..: 290.576232

P-value...............: 0.001

3.Location IDs included.: 621025, 621026, 621024, 621002, 620821, 621021, 620822, 621027,

621023, 620823, 620802, 620881, 621022, 620525, 620825, 620521,

620503, 620826, 621228, 620522, 621227

Coordinates / radius..: (35.405100 N, 108.366000 E) / 273.06 km

Span..................: 336.78 km

Population............: 2274469

Number of cases.......: 1547

Expected cases........: 2315.62

Annual cases / 100000.: 6.2

Observed / expected...: 0.67

Relative risk.........: 0.61

Log likelihood ratio..: 181.418823

P-value...............: 0.001

_______________________________________________________________________________________________

ADDITIONAL RESULTS FILES

Cluster Information : D:\colon cancer\time and space\Middle\high-low.col.txt

Cluster Information : D:\colon cancer\time and space\Middle\high-low.col.dbf

_______________________________________________________________________________________________

PARAMETER SETTINGS

Input

-----

Case File : D:\colon cancer\time and space\Middle\CAS.csv

Population File : D:\colon cancer\time and space\Middle\pop.csv

Time Precision : Year

Start Date : 2013/1/1

End Date : 2023/12/31

Coordinates File : D:\colon cancer\time and space\Middle\geo.csv

Coordinates : Latitude/Longitude

Analysis

--------

Type of Analysis : Purely Spatial

Probability Model : Discrete Poisson

Scan For Areas With : High or Low Rates

Output

------

Main Results File : D:\colon cancer\time and space\Middle\high-low.txt

HTML file for Google Map : No

KML file for Google Earth : No

Shapefile for GIS software : No

HTML file for Cartesian map : No

Cluster Information : Yes (ASCII), Yes (dBase)

Stratified Cluster Information : No (ASCII), No (dBase)

Location Information : No (ASCII), No (dBase)

Risk Estimates for Each Location : No (ASCII), No (dBase)

Simulated Log Likelihood Ratios : No (ASCII), No (dBase)

Data Checking

-------------

Temporal Data Check : Check to ensure that all cases and controls are within the specified temporal study period.

Geographical Data Check : Check to ensure that all observations (cases, controls and populations) are within the specified geographical area.

Spatial Neighbors

-----------------

Specify neighbors through a non-Euclidean neighbors file : No

Specify a meta location file : No

Observations with Multiple Locations : One location per observation.

Locations Network

-----------------

Use Locations Network File : No

Spatial Window

--------------

Maximum Spatial Cluster Size : 25 percent of population at risk

Window Shape : Circular

Isotonic Scan : No

Cluster Restrictions

--------------------

Minimum Cases in Cluster for High Rates : 2

Restrict High Rate Clusters : No

Restrict Low Rate Clusters : No

Space And Time Adjustments

--------------------------

Adjust for Known Relative Risks : No

Inference

---------

P-Value Reporting : Default Combination

Number of Replications : 999

Adjusting for More Likely Clusters : No

Drilldown

---------

Same Design as Main Analysis : No

Miscellaneous Analysis

----------------------

Report Oliveira's F : No

Spatial Output

--------------

Report Hierarchical Clusters : Yes

Criteria for Reporting Secondary Clusters : No Geographical Overlap

Report Gini Optimized Cluster Collection : No

Restrict Reporting to Smaller Clusters : No

Other Output

------------

Report Critical Values : No

Report Monte Carlo Rank : No

Print ASCII Column Headers : No

User Defined Title :

Notifications

-------------

Always Send Email : No

Send Email with Results Meeting Cutoff : No

Run Options

-----------

Processor Usage : All Available Processors

Suppress Warnings : No

Logging Analysis : No

_______________________________________________________________________________________________

RUN INFORMATION

Program completed : Fri Oct 24 21:23:03 2025

Total Running Time : 1 second

Processor Usage : 16 processors
