# Supplementary material for: Integrating geospatial and environmental factors in colorectal cancer epidemiology: a regional study
Source: Front Public Health. 2026 Jan 15;13:1699870. doi: 10.3389/fpubh.2025.1699870 (PMC12852315; doi:10.3389/fpubh.2025.1699870)
Supplement: Supplementary file 7 [file Table_6.docx]

SaTScan v10.3

_____________________________

Program run on: Fri Oct 24 21:39:03 2025

Purely Spatial analysis

scanning for clusters with high or low rates

using the Discrete Poisson model.

_______________________________________________________________________________________________

SUMMARY OF DATA

Study period.......................: 2013/1/1 to 2023/12/31

Number of locations................: 87

Population, averaged over time.....: 3853649

Total number of cases..............: 16689

Annual cases / 100000..............: 39.4

_______________________________________________________________________________________________

CLUSTERS DETECTED

1.Location IDs included.: 620622, 620623, 620602, 620423, 620121, 620402, 620122, 620111,

620621, 620321, 620421, 620104, 620105, 620302, 620403, 620102

Coordinates / radius..: (37.526300 N, 103.331000 E) / 168.43 km

Span..................: 323.64 km

Population............: 915599

Number of cases.......: 6891

Expected cases........: 3965.19

Annual cases / 100000.: 68.4

Observed / expected...: 1.74

Relative risk.........: 2.26

Log likelihood ratio..: 1248.214596

P-value...............: 0.001

2.Location IDs included.: 621223, 623023, 621126, 621226, 621125, 623024, 621225, 620524,

621202, 623021, 620523, 620502, 623022, 621221, 621222, 621122,

621123, 621224, 621227, 621121

Coordinates / radius..: (34.010000 N, 104.443000 E) / 149.40 km

Span..................: 254.53 km

Population............: 930697

Number of cases.......: 1996

Expected cases........: 4030.57

Annual cases / 100000.: 19.5

Observed / expected...: 0.50

Relative risk.........: 0.43

Log likelihood ratio..: 787.244606

P-value...............: 0.001

3.Location IDs included.: 621023, 621021, 621024, 621022, 621002, 621026, 621027, 621025,

620821, 620802, 620823, 620822, 620881, 620825, 620525, 620826,

620521, 620522, 620422

Coordinates / radius..: (36.438000 N, 108.021000 E) / 268.04 km

Span..................: 301.80 km

Population............: 907811

Number of cases.......: 2579

Expected cases........: 3931.46

Annual cases / 100000.: 25.8

Observed / expected...: 0.66

Relative risk.........: 0.59

Log likelihood ratio..: 334.411152

P-value...............: 0.001

_______________________________________________________________________________________________

ADDITIONAL RESULTS FILES

Cluster Information : D:\colon cancer\time and space\old\high-low.col.txt

Cluster Information : D:\colon cancer\time and space\old\high-low.col.dbf

_______________________________________________________________________________________________

PARAMETER SETTINGS

Input

-----

Case File : D:\colon cancer\time and space\old\CAS.csv

Population File : D:\colon cancer\time and space\old\pop.csv

Time Precision : Year

Start Date : 2013/1/1

End Date : 2023/12/31

Coordinates File : D:\colon cancer\time and space\old\geo.csv

Coordinates : Latitude/Longitude

Analysis

--------

Type of Analysis : Purely Spatial

Probability Model : Discrete Poisson

Scan For Areas With : High or Low Rates

Output

------

Main Results File : D:\colon cancer\time and space\old\high-low.txt

HTML file for Google Map : No

KML file for Google Earth : No

Shapefile for GIS software : No

HTML file for Cartesian map : No

Cluster Information : Yes (ASCII), Yes (dBase)

Stratified Cluster Information : No (ASCII), No (dBase)

Location Information : No (ASCII), No (dBase)

Risk Estimates for Each Location : No (ASCII), No (dBase)

Simulated Log Likelihood Ratios : No (ASCII), No (dBase)

Data Checking

-------------

Temporal Data Check : Check to ensure that all cases and controls are within the specified temporal study period.

Geographical Data Check : Check to ensure that all observations (cases, controls and populations) are within the specified geographical area.

Spatial Neighbors

-----------------

Specify neighbors through a non-Euclidean neighbors file : No

Specify a meta location file : No

Observations with Multiple Locations : One location per observation.

Locations Network

-----------------

Use Locations Network File : No

Spatial Window

--------------

Maximum Spatial Cluster Size : 25 percent of population at risk

Window Shape : Circular

Isotonic Scan : No

Cluster Restrictions

--------------------

Minimum Cases in Cluster for High Rates : 2

Restrict High Rate Clusters : No

Restrict Low Rate Clusters : No

Space And Time Adjustments

--------------------------

Adjust for Known Relative Risks : No

Inference

---------

P-Value Reporting : Default Combination

Number of Replications : 999

Adjusting for More Likely Clusters : No

Drilldown

---------

Same Design as Main Analysis : No

Miscellaneous Analysis

----------------------

Report Oliveira's F : No

Spatial Output

--------------

Report Hierarchical Clusters : Yes

Criteria for Reporting Secondary Clusters : No Geographical Overlap

Report Gini Optimized Cluster Collection : No

Restrict Reporting to Smaller Clusters : No

Other Output

------------

Report Critical Values : No

Report Monte Carlo Rank : No

Print ASCII Column Headers : No

User Defined Title :

Notifications

-------------

Always Send Email : No

Send Email with Results Meeting Cutoff : No

Run Options

-----------

Processor Usage : All Available Processors

Suppress Warnings : No

Logging Analysis : No

_______________________________________________________________________________________________

RUN INFORMATION

Program completed : Fri Oct 24 21:39:03 2025

Total Running Time : 0 seconds

Processor Usage : 16 processors
