# Supplementary material for: Integrating geospatial and environmental factors in colorectal cancer epidemiology: a regional study
Source: Front Public Health. 2026 Jan 15;13:1699870. doi: 10.3389/fpubh.2025.1699870 (PMC12852315; doi:10.3389/fpubh.2025.1699870)
Supplement: Supplementary file 8 [file Table_7.docx]

SaTScan v10.3

_____________________________

Program run on: Fri Oct 24 19:06:20 2025

Retrospective Space-Time analysis

scanning for clusters with high or low rates

using the Discrete Poisson model.

_______________________________________________________________________________________________

SUMMARY OF DATA

Study period.......................: 2013/1/1 to 2023/12/31

Number of locations................: 87

Population, averaged over time.....: 25559425

Total number of cases..............: 27516

Annual cases / 100000..............: 9.8

_______________________________________________________________________________________________

CLUSTERS DETECTED

1.Location IDs included.: 623024, 623022, 621126, 623021, 621223, 623023, 621125, 623026,

623001, 621123, 622922, 621226, 620524, 622925, 623027, 621122,

621222, 622924, 621225, 621202, 621124, 622921, 623025

Coordinates / radius..: (34.004700 N, 103.562000 E) / 175.74 km

Span..................: 337.96 km

Time frame............: 2013/1/1 to 2017/12/31

Population............: 5901636

Number of cases.......: 602

Expected cases........: 2942.92

Annual cases / 100000.: 2.0

Observed / expected...: 0.20

Relative risk.........: 0.19

Log likelihood ratio..: 1493.724922

P-value...............: 0.001

2.Location IDs included.: 620102

Coordinates / radius..: (36.074300 N, 103.874000 E) / 0 km

Span..................: 0 km

Time frame............: 2013/1/1 to 2017/12/31

Population............: 1363921

Number of cases.......: 2119

Expected cases........: 630.17

Annual cases / 100000.: 32.9

Observed / expected...: 3.36

Relative risk.........: 3.56

Log likelihood ratio..: 1122.901133

P-value...............: 0.001

3.Location IDs included.: 620302, 620321, 620725, 620621, 620602, 620722, 620702, 620622,

620623, 620723, 620423, 620724, 620121, 620721, 620111, 620402,

620122, 620104, 622923, 620902, 620421

Coordinates / radius..: (38.645400 N, 102.158000 E) / 309.99 km

Span..................: 608.40 km

Time frame............: 2019/1/1 to 2023/12/31

Population............: 5967044

Number of cases.......: 5218

Expected cases........: 2783.56

Annual cases / 100000.: 18.3

Observed / expected...: 1.87

Relative risk.........: 2.08

Log likelihood ratio..: 968.418334

P-value...............: 0.001

4.Location IDs included.: 621024, 621026, 621023, 621021, 621025, 621002, 621027, 620821,

621022, 620822, 620823

Coordinates / radius..: (36.001900 N, 108.299000 E) / 144.87 km

Span..................: 177.81 km

Time frame............: 2013/1/1 to 2017/12/31

Population............: 2748158

Number of cases.......: 271

Expected cases........: 1370.36

Annual cases / 100000.: 1.9

Observed / expected...: 0.20

Relative risk.........: 0.19

Log likelihood ratio..: 682.946995

P-value...............: 0.001

5.Location IDs included.: 620826, 620825, 621121, 620522, 620525, 620422, 620523, 620521

Coordinates / radius..: (35.429400 N, 105.667000 E) / 87.37 km

Span..................: 164.65 km

Time frame............: 2013/1/1 to 2017/12/31

Population............: 3192397

Number of cases.......: 578

Expected cases........: 1657.09

Annual cases / 100000.: 3.4

Observed / expected...: 0.35

Relative risk.........: 0.33

Log likelihood ratio..: 492.519858

P-value...............: 0.001

6.Location IDs included.: 620902, 620200, 620724

Coordinates / radius..: (39.603400 N, 98.791500 E) / 69.01 km

Span..................: 122.61 km

Time frame............: 2013/1/1 to 2017/12/31

Population............: 851619

Number of cases.......: 1059

Expected cases........: 402.51

Annual cases / 100000.: 25.8

Observed / expected...: 2.63

Relative risk.........: 2.70

Log likelihood ratio..: 375.962431

P-value...............: 0.001

7.Location IDs included.: 620302

Coordinates / radius..: (38.645400 N, 102.158000 E) / 0 km

Span..................: 0 km

Time frame............: 2013/1/1 to 2017/12/31

Population............: 241162

Number of cases.......: 466

Expected cases........: 112.34

Annual cases / 100000.: 40.6

Observed / expected...: 4.15

Relative risk.........: 4.20

Log likelihood ratio..: 311.596433

P-value...............: 0.001

8.Location IDs included.: 623024, 623022, 621126, 623021, 621223, 623023, 621125, 623026,

623001, 621123, 622922, 621226, 620524, 622925, 623027, 621122,

621222, 622924, 621225

Coordinates / radius..: (34.004700 N, 103.562000 E) / 163.18 km

Span..................: 311.91 km

Time frame............: 2019/1/1 to 2022/12/31

Population............: 4455444

Number of cases.......: 1105

Expected cases........: 1701.36

Annual cases / 100000.: 6.4

Observed / expected...: 0.65

Relative risk.........: 0.63

Log likelihood ratio..: 126.298785

P-value...............: 0.001

9.Location IDs included.: 620881

Coordinates / radius..: (35.198500 N, 106.595000 E) / 0 km

Span..................: 0 km

Time frame............: 2019/1/1 to 2022/12/31

Population............: 188156

Number of cases.......: 9

Expected cases........: 72.87

Annual cases / 100000.: 1.2

Observed / expected...: 0.12

Relative risk.........: 0.12

Log likelihood ratio..: 45.122889

P-value...............: 0.001

10.Location IDs included.: 621002, 620821

Coordinates / radius..: (35.669100 N, 107.662000 E) / 41.27 km

Span..................: 41.27 km

Time frame............: 2020/1/1 to 2022/12/31

Population............: 690153

Number of cases.......: 339

Expected cases........: 215.25

Annual cases / 100000.: 15.4

Observed / expected...: 1.57

Relative risk.........: 1.58

Log likelihood ratio..: 30.506548

P-value...............: 0.001

11.Location IDs included.: 621023, 621021, 621024

Coordinates / radius..: (36.438000 N, 108.021000 E) / 54.49 km

Span..................: 56.52 km

Time frame............: 2019/1/1 to 2022/12/31

Population............: 519363

Number of cases.......: 108

Expected cases........: 197.52

Annual cases / 100000.: 5.4

Observed / expected...: 0.55

Relative risk.........: 0.54

Log likelihood ratio..: 24.466876

P-value...............: 0.001

12.Location IDs included.: 621025

Coordinates / radius..: (35.405100 N, 108.366000 E) / 0 km

Span..................: 0 km

Time frame............: 2019/1/1 to 2021/12/31

Population............: 179650

Number of cases.......: 18

Expected cases........: 52.00

Annual cases / 100000.: 3.4

Observed / expected...: 0.35

Relative risk.........: 0.35

Log likelihood ratio..: 14.925436

P-value...............: 0.001

13.Location IDs included.: 620802

Coordinates / radius..: (35.510900 N, 106.738000 E) / 0 km

Span..................: 0 km

Time frame............: 2019/1/1 to 2019/12/31

Population............: 509122

Number of cases.......: 88

Expected cases........: 51.55

Annual cases / 100000.: 16.7

Observed / expected...: 1.71

Relative risk.........: 1.71

Log likelihood ratio..: 10.636573

P-value...............: 0.065

14.Location IDs included.: 620823

Coordinates / radius..: (35.245200 N, 106.993000 E) / 0 km

Span..................: 0 km

Time frame............: 2019/1/1 to 2021/12/31

Population............: 96042

Number of cases.......: 51

Expected cases........: 26.23

Annual cases / 100000.: 19.0

Observed / expected...: 1.94

Relative risk.........: 1.95

Log likelihood ratio..: 9.155511

P-value...............: 0.211

_______________________________________________________________________________________________

ADDITIONAL RESULTS FILES

Cluster Information : D:\colon cancer\time and space\mingan-shikong\high-low-50.col.txt

Cluster Information : D:\colon cancer\time and space\mingan-shikong\high-low-50.col.dbf

_______________________________________________________________________________________________

PARAMETER SETTINGS

Input

-----

Case File : D:\colon cancer\time and space\All\CAS.csv

Population File : D:\colon cancer\time and space\All\pop.csv

Time Precision : Year

Start Date : 2013/1/1

End Date : 2023/12/31

Coordinates File : D:\colon cancer\time and space\All\geo.csv

Coordinates : Latitude/Longitude

Analysis

--------

Type of Analysis : Retrospective Space-Time

Probability Model : Discrete Poisson

Scan For Areas With : High or Low Rates

Time Aggregation Units : Year

Time Aggregation Length : 1

Output

------

Main Results File : D:\colon cancer\time and space\mingan-shikong\high-low-50.txt

HTML file for Google Map : No

KML file for Google Earth : No

Shapefile for GIS software : No

HTML file for Cartesian map : No

Cluster Information : Yes (ASCII), Yes (dBase)

Stratified Cluster Information : No (ASCII), No (dBase)

Location Information : No (ASCII), No (dBase)

Risk Estimates for Each Location : No (ASCII), No (dBase)

Simulated Log Likelihood Ratios : No (ASCII), No (dBase)

Data Checking

-------------

Temporal Data Check : Check to ensure that all cases and controls are within the specified temporal study period.

Geographical Data Check : Check to ensure that all observations (cases, controls and populations) are within the specified geographical area.

Spatial Neighbors

-----------------

Specify neighbors through a non-Euclidean neighbors file : No

Specify a meta location file : No

Observations with Multiple Locations : One location per observation.

Locations Network

-----------------

Use Locations Network File : No

Spatial Window

--------------

Maximum Spatial Cluster Size : 25 percent of population at risk

Include Purely Temporal Clusters : No

Window Shape : Circular

Temporal Window

---------------

Minimum Temporal Cluster Size : 1 Year

Maximum Temporal Cluster Size : 50 percent of study period

Include Purely Spatial Clusters : No

Cluster Restrictions

--------------------

Minimum Cases in Cluster for High Rates : 2

Restrict High Rate Clusters : No

Restrict Low Rate Clusters : No

Space And Time Adjustments

--------------------------

Temporal Adjustment : None

Adjust for Weekly Trends, Nonparametric : No

Spatial Adjustment : None

Adjust for Known Relative Risks : No

Inference

---------

P-Value Reporting : Default Combination

Number of Replications : 999

Adjusting for More Likely Clusters : No

Drilldown

---------

Same Design as Main Analysis : No

Purely Spatial Bernoulli : No

Spatial Output

--------------

Report Hierarchical Clusters : Yes

Criteria for Reporting Secondary Clusters : No Geographical Overlap

Restrict Reporting to Smaller Clusters : No

Temporal Graphs

---------------

Produce Temporal Graphs : No

Other Output

------------

Report Critical Values : No

Report Monte Carlo Rank : No

Print ASCII Column Headers : No

User Defined Title :

Notifications

-------------

Always Send Email : No

Send Email with Results Meeting Cutoff : No

Run Options

-----------

Processor Usage : All Available Processors

Suppress Warnings : No

Logging Analysis : No

_______________________________________________________________________________________________

RUN INFORMATION

Program completed : Fri Oct 24 19:06:20 2025

Total Running Time : 0 seconds

Processor Usage : 16 processors
